# snRNA 3′ End Processing by a CPSF73-Containing Complex Essential for Development in *Arabidopsis*

**DOI:** 10.1371/journal.pbio.1002571

**Published:** 2016-10-25

**Authors:** Yunfeng Liu, Shengjun Li, Yuan Chen, Athen N. Kimberlin, Edgar B. Cahoon, Bin Yu

**Affiliations:** 1 Center for Plant Science Innovation and School of Biological Sciences, University of Nebraska, Lincoln, Nebraska, United States of America; 2 Plant Gene Expression Center, US Department of Agriculture-Agricultural Research Service, University of California-Berkeley, Albany, California, United States of America; 3 Center for Plant Science Innovation and Department of Biochemistry, University of Nebraska, Lincoln, Nebraska, United States of America; Max Planck Institute for Developmental Biology, GERMANY

## Abstract

Uridine-rich small nuclear RNAs (snRNAs) are the basal components of the spliceosome and play essential roles in splicing. The biogenesis of the majority of snRNAs involves 3′ end endonucleolytic cleavage of the nascent transcript from the elongating DNA-dependent RNA ploymerase II. However, the protein factors responsible for this process remain elusive in plants. Here, we show that DEFECTIVE in snRNA PROCESSING 1 (DSP1) is an essential protein for snRNA 3′ end maturation in *Arabidopsis*. A hypomorphic *dsp1-1* mutation causes pleiotropic developmental defects, impairs the 3′ end processing of snRNAs, increases the levels of snRNA primary transcripts (pre-snRNAs), and alters the occupancy of Pol II at snRNA loci. In addition, DSP1 binds snRNA loci and interacts with Pol-II in a DNA/RNA-dependent manner. We further show that DSP1 forms a conserved complex, which contains at least four additional proteins, to catalyze snRNA 3′ end maturation in *Arabidopsis*. The catalytic component of this complex is likely the cleavage and polyadenylation specificity factor 73 kDa-I (CSPF73-I), which is the nuclease cleaving the pre-mRNA 3′ end. However, the DSP1 complex does not affect pre-mRNA 3′ end cleavage, suggesting that plants may use different CPSF73-I-containing complexes to process snRNAs and pre-mRNAs. This study identifies a complex responsible for the snRNA 3′ end maturation in plants and uncovers a previously unknown function of CPSF73 in snRNA maturation.

## Abstract 2

This study identifies a protein complex in plants that is responsible for the maturation of the 3′ ends of spliceosomal snRNAs and uncovers a novel function for the mRNA 3′ cleavage nuclease CPSF73.

## Author Summary

snRNAs form the RNA components of the spliceosome and are required for spliceosome formation and splicing. The generation of snRNAs involves 3′ end endonucleolytic cleavage of primary snRNA transcripts (pre-snRNAs). The factors responsible for pre-snRNA 3′ end cleavage are known in metazoans, but many of these components are missing in plants. Therefore, the proteins that catalyze pre-snRNA cleavage in plants and the mechanism leading to plant snRNA 3′ maturation are unknown. Here, we show that a DSP1 complex (containing DSP1, DSP2, DSP3, DSP4, and CPFS73-I) is responsible for pre-snRNA 3′ end cleavage in *Arabidopsis*. We further show that CPSF73-I, which is known to cleave the pre-mRNA 3′ end, is likely the enzyme also catalyzing snRNA 3′ end maturation in plants. Interestingly, plants appear to use two different CPSF73-I-containing complexes to catalyze the maturation of mRNAs and snRNAs. The study thereby identifies an snRNA-processing complex in plants and also elucidates a new role for CPSF73-I in this process.

## Introduction

Uridine-rich small nuclear RNAs (snRNAs), ~60–200 nucleotide (nt) in length, are conserved noncoding RNAs in eukaryotes [1,2]. As the RNA components of the spliceosome, snRNAs (U1, U2, U4, U5, and U6) play essential roles in spliceosome formation and splicing of pre-messenger RNAs (pre-mRNAs) [1–3]. Most snRNAs are derived from their primary transcripts (pre-snRNAs) generated by DNA-dependent RNA polymerase II (Pol II), with the exception of Pol III-dependent U6 [4–7]. Like pre-mRNAs, pre-snRNAs are transcribed beyond the 3′ end of mature snRNAs [4,8,9]. Consequently, pre-snRNAs subject to 3′ maturation, a process involving endonucleolytic cleavage of the nascent transcript from the elongating polymerase in the nucleus followed by a 3′-to-5′ exonucleolytic trimming step in the cytoplasm [4,8,9].

Previous studies have identified three elements required for proper 3′ end cleavage of Pol II-dependent snRNAs in metazoans: an snRNA promoter containing the distal sequence element (DSE) and the proximal sequence element (PSE), the C-terminal domain (CTD) of Rpb1 (the largest subunit of Pol II), and the 3′ box that localizes the downstream of the cleavage site [5,8–13]. In metazoans, the integrator complex (INT), which contains at least 14 subunits, is responsible for pre-snRNA 3′ end cleavage [14]. Among INT subunits, INT1, 4, 9, and 11 are essential for snRNA 3′ processing, whereas INT3 and 10 are dispensable for maturation [14,15]. INT11 is a paralog of the cleavage and polyadenylation specificity factor 73 kDa (CPSF73), which is the catalytic component of the CPSF complex that cleaves mRNAs, but not snRNAs, at the 3′ end [14]. Because of this, INT11 was proposed to cleave pre-snRNA at 3′ end [14,16]. INT requires Pol II and the promoter elements for its recruitment to snRNA loci [6,17–21]. However, it is not clear how INT specifically recognizes snRNA loci and transcripts. Yeast uses different mechanisms to process the snRNA 3′ end because it does not contain INT, and its snRNA gene structures differ from their metazoan counterparts [6,7].

In plants, the major Pol II-dependent snRNAs include U1, U2, U4, and U5 [22–29]. Each of them has more than ten copies in the *Arabidopsis* genome [30]. Although plant snRNA promoters have diverged from their metazoan counterparts and do not contain DSE and PSE, they do have an upstream sequence element (USE) and a proximal TATA box, which are conserved and essential for their transcription [25]. Plant snRNA genes have a conserved 3′ box (CA (N)3-10AGTNNAA) downstream of mature snRNAs, which is necessary for snRNA processing [27,31]. In plants, the processing of snRNAs can be uncoupled from transcription initiation, because their promoters are not required for 3′ end cleavage [31]. In addition, many subunits of INT, including INT11 and the putative scaffold protein INT1, are missing in plants [16], suggesting that plants may use a mechanism different from that of metazoans to process snRNA 3′ end.

Here, we report that snRNA 3′ end maturation in *Arabidopsis* requires a protein named DEFECTIVE in snRNA PROCESSING 1 (DSP1). DSP1 binds snRNA loci and interacts with Pol II in a DNA/RNA-dependent manner. A hypomorphic *dsp1-1* mutation causes pleiotropic developmental defects, impairs snRNA 3′ maturation, and alters the occupancy of Pol II at snRNA loci. DSP1 forms a conserved complex with DSP2, DSP3, DSP4, and CPSF73-I to process snRNAs. Unlike CPSF73-I, which is also the catalytic component of the plant CPSF complex, the DSP1 complex does not affect mRNA 3′ maturation. Based on these results, we propose that two CPSF73-I complexes separately process pre-snRNAs and pre-mRNAs in *Arabidopsis*. This study identifies an snRNA-processing complex and uncovers an unknown function for CPSF73 in plants.

## Results

### Identification of a Mutant Deficient in U2 snRNA Biogenesis

**Fig 1:**
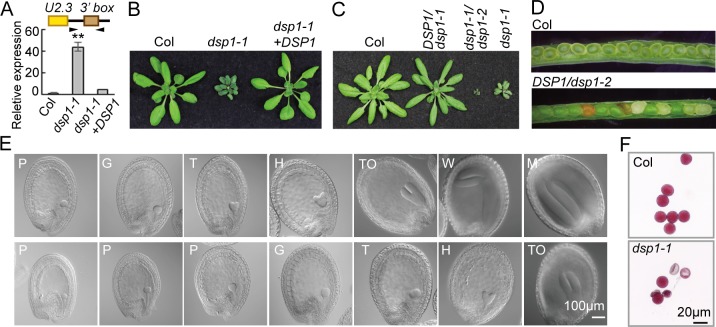
*dsp1-1* increases the abundance of *pre-U2*.*3* snRNAs and causes pleiotropic developmental defects. **(A)** The accumulation of *pre-U2*.*3* snRNAs detected by quantitative RT-PCR (qRT-PCR). *dsp1-1*+*DSP1*: *dsp1-1* harboring a WT copy of *DSP1*; Col: WT. The levels of *pre-U2*.*3* snRNAs in *dsp1-1* were normalized to those of *UBIQUITIN 5* (*UBQ5*) and compared with Col. Error bars indicate standard deviation (SD) of three technical replicates (**p < 0.01). Similar results were obtained from three biological replicates. Black arrowheads: primers used for RT-PCR; Black arrow: cleavage sites. **(B)** and **(C)** Morphological phenotypes of various genotypes. **(D)** Dissected green siliques of Col and *DSP1/dsp1-2*. **(E)** Embryo development of Col (top row) and *dsp1-1* (bottom row). Embryos from siliques that were pollinated and harvested at the same time are shown in one column for comparison (Col versus *dsp1-1*). Embryo stages: preglobular (P), globular (G), transition (T), heart (H), torpedo (To), walking-stick (W), and mature embryo (M). **(F)** Pollen viability of Col and *dsp1-1*. Viable pollens are purple and round, whereas defective pollens are less stained. Bars = 100 μm (E) and 20 μm (F).

In order to identify proteins involved in snRNA maturation in *Arabidopsis*, we screened for mutants containing increased levels of *pre-U2*.*3* snRNA (At3g57765) from a T-DNA collection obtained from the *Arabidopsis* Stock Center. We reasoned that impaired snRNA 3′ end cleavage should increase the levels of pre-snRNAs. From ~ 500 T-DNA insertion lines, we identified a mutant (Salk_036641C) containing elevated levels of *pre-U2*.*3* snRNA relative to wild-type plants (WT; Columbia-0 [Col]) through reverse transcription PCR (RT-PCR) analyses (Fig 1A and S1 Data). We named this mutant *defective in snRNA processing 1–1* (*dsp1-1*). In *dsp1-1*, a T-DNA insertion in the second intron of At4g20060 (*DSP1*) reduced the transcript levels of *DSP1* (S1A–S1C Fig). However, *dsp1-1* showed incomplete penetrance, as only a portion of plants showed increased levels of *pre-U2*.*3* snRNA, accompanied with pleiotropic development defects such as smaller size, delayed flowering, reduced fertility, and enlarged cell size (Fig 1B and S1D–S1F Fig). To demonstrate that *dsp1-1* is responsible for the observed phenotypes, we crossed *dsp1-1* to *DSP1*/*dsp1-2* (CS16199), which contains a T-DNA insertion in the sixth exon of *DSP1* (S1A and S1B Fig). The F1 *dsp1-1/dsp1-2* mutant displayed more severe growth defects and higher levels of *pre-U2*.*3* snRNA than *dsp1-1* (Fig 1C and S1G Fig, S1 Data). Furthermore, a WT copy of *DSP1* driven by its native promoter (*pDSP1*::*DSP1-Green Fluorescent Protein* [*GFP*]) in *dsp1-1* rescued the developmental defects and restored the levels of *pre-U2*.*3* snRNA (Fig 1A and 1B, S1 Data), demonstrating that DSP1 is required for plant development and may be involved in snRNA biogenesis.

### DSP1 Is Required for Pollen Viability and Embryogenesis

We suspected that the *dsp1-2* mutation might cause embryo lethality, because the homozygous *dsp1-2* mutant could not be obtained, and aborted seeds were observed in siliques of *DSP1/dsp1-2* (Fig 1D). In fact, Nomarski microscopy showed embryos, whose terminal phenotype arrested at the globular stage, in the siliques of *DSP1*/*dsp1-2* (S1H Fig). Agreeing with this result, most *dsp1-1/dsp1-1* seeds displayed delayed embryo development relative to WT (Fig 1E). Furthermore, a small portion of *dsp1-1* seeds contained abnormal embryos (S1I Fig), suggesting that *dsp1-1* might impair cell division and/or pattern formation.

We also found that the transmission of *dsp1-2* was reduced, as the ratio of *DSP1/dsp1-2* versus WT (1:1.3) was less than the expected ratio (1:1) in offspring of *DSP1/dsp1-2*. To determine whether DSP1 influences male or female gametophyte transmission, we performed reciprocal crosses between *DSP1*/*dsp1-2* and WT and analyzed transmission of *dsp1-2*. When WT was used as a pollen donor, *dsp1-2* was transmitted normally (S1 Table). However, when *DSP1/dsp1-2* was used as a pollen donor, the transmission rate of *dsp1-2* was reduced (S1 Table), suggesting that *dsp1-2* might affect male gametophyte transmission. In order to examine how *dsp1* influences male gametophyte transmission, we first examined pollen viability using Alexander's staining. Although pollens from WT appeared full, round, and red-stained, many pollens from *dsp1-1* could not be stained (Fig 1F), suggesting that they are completely or partially devoid of cytoplasmic content, indicative of a defect in pollen viability. We also examined pollen germination and tube growth of the viable *dsp1-1* pollen grains but did not observe obvious differences from WT (S1J and S1K Fig). These results suggest that DSP1 participates in male gametophyte transmission by influencing pollen viability.

### DSP1 Is Required for snRNA Processing

**Fig 2:**
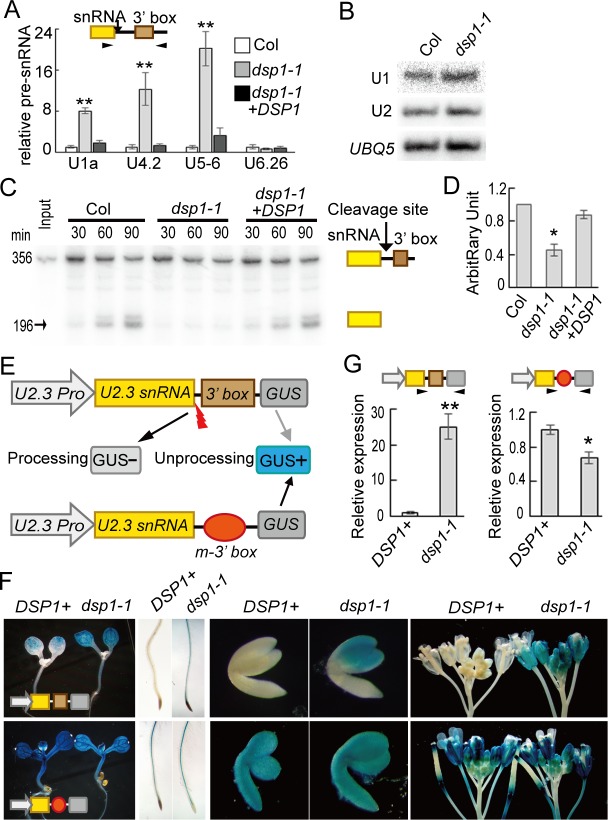
DSP1 is required for snRNA 3′ end maturation. **(A)** The accumulation of various pre-snRNAs detected by qRT-PCR. The levels of pre-snRNAs in *dsp1-1* were normalized to those of *UBQ5* and compared with Col. Error bars indicate SD of three technical replications (**p < 0.01). Black arrowheads: primers used for RT-PCR; Black arrow: cleavage sites. **(B)** The accumulation of mature U1 and U2 snRNAs detected by northern Blot. *UBQ5* was blotted as a loading control. **(C)** In vitro processing of *pre-U2*.*3* snRNAs by nuclear protein extracts from Col (WT) and *dsp1-1*. Arrow indicates mature snRNAs. **(D)** Quantification of mature *U2*.*3* RNA production in *dsp1-1* relative to Col. The reaction stopped at 90 min was used for quantification analysis. The radioactive signal of mature *U2*.*3* RNAs was quantified with Quantity One and then normalized to input. The relative levels of mature *U2* snRNAs produced by *dsp1-1* were then compared with those produced by Col. The value of Col was set to 1. The value represents the mean of three repeats. *p < 0.05 (*t* test). **(E)** Diagram of the *pU2*::*pre-U2-GUS* and *pU2*::*pre-U2m-GUS* transgenes. GUS+: GUS protein was produced. GUS-: no GUS protein was produced. **(F)** GUS levels detected by histochemical staining in various tissues of transgenic plants harboring *pU2*::*pre-U2-GUS* (Top row) or *pU2*::*pre-U2m-GUS* (Bottom row). Twenty plants were analyzed. A representative image is shown in each case. *DSP*^*+*^: *DSP1/DSP1* or *DSP1/dsp1-1*. **(G)** The expression of *pre-U2-GUS* RNAs and *pre-U2m-GUS* RNAs in *DSP*^*+*^ and *dsp1-1* detected by RT-PCR. Black arrowheads: primers used for PCR. The levels of *pre-U2-GUS/pre-U2m-GUS* were normalized to *UBQ5* and compared with those in *DSP*^*+*^ (value set to 1). RNAs extracted from inflorescences were used for qRT-PCR analyses.

Because the structures of Pol II-dependent snRNA genes share considerable similarities [30], we hypothesized that DSP1 might have a general effect on pre-snRNA levels. To test this hypothesis, we randomly selected several Pol II-dependent pre-snRNAs from the U1, U4, and U5 gene families and examined their abundance in WT and *dsp1-1* by qRT-PCR and RT-PCR. The accumulation of these selected *pre-snRNAs* (*pre-U1a*, *pre-U2*.*3*, *pre-U4*.*2*, and *pre-U5*.*6* snRNAs) was much higher in *dsp1-1* than that in WT, which was rescued by the *GFP-DSP1* transgene (Fig 2A and S2A Fig, S1 Data). In contrast, the abundance of Pol III-dependent *pre-U6*.*26* snRNA was not affected by *dsp1-1* (Fig 2A and S2A Fig, S1 Data). These results suggest that DSP1 likely has a general role in the biogenesis of Pol II-dependent snRNAs.

We further examined the effect of *dsp1-1* on the accumulation of mature U1 and U2 snRNAs using northern blot. As observed in metazoans [14], the abundance of mature U1 and U2 RNAs in *dsp1-1* was comparable to that in WT (Fig 2B), which could be explained by the facts that *dsp1-1* is a hypomorphic mutation and snRNAs have a long half-life [32]. Cloning and sequencing analyses further showed that mature U2 RNAs were proper processing products (S2B Fig). RNase protection assay showed the increased accumulation of *pre-U1* and *pre-U2* snRNAs in *dsp1-1* and confirmed the results obtained from northern blot (S2C and S2D Fig). Consistent with its effect on mature snRNAs, *dsp1-1* did not impact the splicing of several examined mRNAs (S2E Fig).

The increased *pre-snRNA* levels in *dsp1-1* could result from defection in pre-snRNA 3′ end cleavage or increased *pre-snRNA* transcription. To distinguish these two possibilities, we first evaluated if *dsp1-1* influenced *pre-U2*.*3* snRNA 3′ end cleavage with an in vitro assay using the *U2*.*3* gene as reporter according to [13]. In this assay, a 5′ end [P^32^]-labeled *pre-U2*.*3* snRNA was processed in nuclear proteins extracted from inflorescences of *dsp1-1* or WT. We also included a *pre-U2*.*3* snRNA with a poly-G tail at 3′ end (*pre-U2*.*3-pG)*, which prevents 3′ trimming activity [33], to rule out the possibility that the product is generated from the 3′ end trimming rather than endonucleolytic cleavage. The accumulation of *U2*.*3* snRNAs (~196 nt) generated from both *pre-U2*.*3* and *pre-U2*.*3-pG* was reduced in *dsp1-1* relative to their levels in WT at various time points (Fig 2C and S2F Fig). Quantification analysis of the 90-min reaction showed that the overall *pre-U2*.*3* snRNA processing activity in *dsp1-1* was approximately 40% of that in WT (Fig 2D and S1 Data). In addition, the *DSP1-GFP* transgene restored *pre-U2*.*3* snRNA processing in *dsp1-1* (Fig 2C and 2D, S1 Data). These results suggest that DSP1 might be required for the snRNA 3′ end maturation.

To further test the effect of DSP1 on snRNA transcription and 3′ maturation, we used an in vivo *GUS* reporter gene assay. In this assay, the *GUS* gene was fused to the 3′ end of the *U2*.*3* gene that contains the promoter, the coding region, and 3′ box region (*pU2*::*pre-U2-GUS*; Fig 2E) according to [15]. If properly cleaved, *pre-U2-GUS* RNAs would not be translated into GUS protein (Fig 2E), whereas disrupted cleavage would result in GUS accumulation. As a control for transcription, we generated a *GUS* reporter fused with a mutated *U2*.*3* gene (*pU2*::*pre-U2m-GUS*), in which the 3′ box was mutated to disrupt pre-U2 snRNA processing (Fig 2F), with expectation that the GUS protein would be accumulated (Fig 2E and S2G and S2H Fig). The alteration of *pre-U2m-GUS* levels in *dsp1-1* relative to WT would reflect the effect of DSP1 on pre-snRNA other than cleavage. Transgenic lines expressing *pU2*::*pre-U2-GUS* or *pU2*::*pre-U2m-GUS* were generated in a Col background and subsequently crossed to *dsp1-1* (S2H Fig). In F2, *DSP1*^+^ (*DSP1*/*DSP1* or *DSP1*/*dsp1-1*), or *dsp1-1*, genotypes containing the *GUS* transgene were identified through PCR genotyping. GUS activities and the abundance of *pre-U2m-GUS* transcripts were slightly reduced in *dsp1-1* relative to *DSP1*^+^ (Fig 2F and 2G and S2I Fig, S1 Data; bottom panel), suggesting that *dsp1-1* does not increase the transcription of pre-snRNAs. In contrast, relative to *DSP1*^+^, the GUS activities and *pre-U2-GUS* transcript levels were increased in various tissues of *dsp1-1* harboring *pre-U2-GUS* (Fig 2F and 2G and S2I Fig, S1 Data; top panel). These results demonstrate that DSP1 is essential for snRNA 3′ end cleavage.

### DSP1 Associates with snRNA Loci

**Fig 3:**
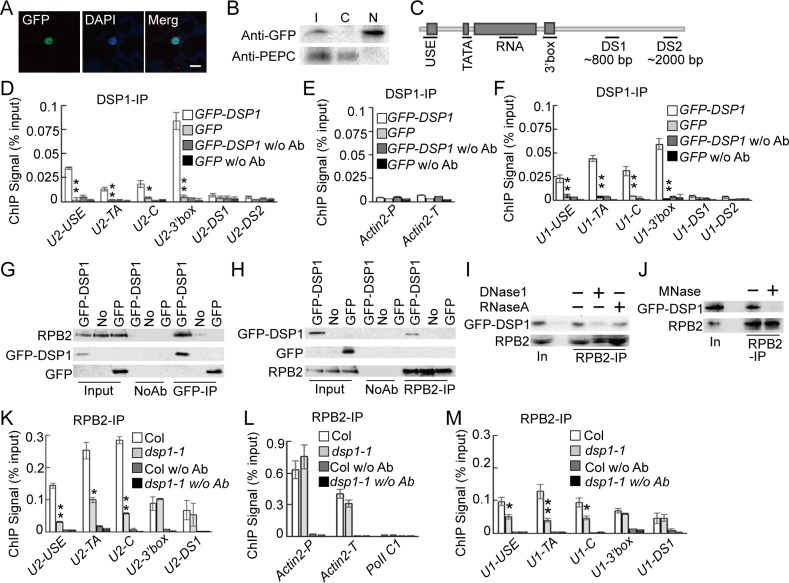
DSP1 binds the snRNA loci and affects the occupancy of Pol II at the snRNA loci. **(A)** Subcellular localization of DSP1-GFP in tobacco leaf epidermal cells. The nuclei were visualized by DAPI staining of DNA. Scale bars: 20 μm. **(B)** Detection of DSP1-GFP in the nuclear and cytoplasmic protein fractions. T: Total proteins; N: Nuclear fraction; C: Cytoplasmic fraction. PEPC: the cytoplasm-localized phosphoenolpyruvate carboxylase. (**C)** Diagram showing the regions within the *U2*.*3* and *U1a* loci detected by ChIP. **(D–F)** The occupancy of DSP1 at various loci in the cotyledons of plants harboring *DSP1-GFP* or *GFP*. *Actin2-P*: the promoter region of *ACTIN2*; *Actin2-T*: the coding region of *ACTIN2*. DNAs co-purified with DSP1-GFP or GFP were analyzed using quantitative PCR (qPCR). Means and SDs of three technical repeats are presented. Three biological replicates gave similar results. **p < 0.01, * *p* < 0.05 (*t* test). **(G)** and **(H)** Co-immunoprecipitation (Co-IP) between GFP-DSP1 and Pol II. **(I)** and **(J)** Co-IP between GFP-DSP1 and Pol II requires both DNA and RNA. Transgenic plants or nontransgenic plants (No) were indicated on Top. Proteins detected by western blot are labeled on the left. In: Input; MNase: Micrococcal Nuclease. **(K–M)** The occupancy of Pol II at various DNA loci in Col and *dsp1-1*. *Actin2-P*: the promoter region of *ACTIN2*; *Actin2-T*: the coding region of *ACTIN2*. DNAs co-purified with Pol II were analyzed using qPCR. Pol II C1 is used as a negative control for Pol II occupancy. Means and SDs of three technical repeats are presented. Similar results were obtained from three biological replicates. **p < 0.01, *p < 0.05 (*t* test).

In metazoans, the INT complex co-transcriptionally processes pre-snRNAs [16]. This led us to hypothesize that DSP1, if it has a direct role in snRNA processing, might be a nuclear-localized protein that associates with the snRNA loci. To examine the subcellular localization of DSP1, we expressed *GFP-DSP1* from the CaMV35S promoter *(35S*::*GFP-DSP1)* in leaf epidermal cells of *Nicotiana benthamiana*. In these cells, GFP-DSP1 localized to the nucleus (Fig 3A). Consistent with this result, GFP-DSP1 was detected in the nuclear protein fraction, but not in the cytoplasmic protein fraction (Fig 3B), both of which were extracted from the *dsp1-1* harboring *35S*::*GFP-DSP1*.

To examine the association of DSP1 with the *U2*.*3* locus, we performed a chromatin immunoprecipitation (ChIP) assay using *dsp1-1* harboring *GFP-DSP1* or GFP (negative control) and then checked the presence of the *U2*.*3* locus in the ChIPs of GFP-DSP1 and GFP IPs using PCR and quantitative PCR (qPCR). The USE, TATA box (U2-TA), coding region (U2-C), and 3′ box (U2-3′ box; the highest signal) of the *U2*.*3* locus were enriched in the ChIPs of GFP-DSP1, but not in the ChIPs for GFP, relative to “no-antibody” controls (Fig 3C and 3D and S3A Fig, S1 Data). In addition, the downstream regions (U2-DS1 and U2-DS2) of the 3′ box of the *U2*.*3* locus and the *ACTIN2* locus (Pol II-dependent) were not enriched in the ChIPs of GFP-DSP1 (Fig 3C–3E and S3A and S3B Fig, S1 Data). DSP1 also occupied the USE, TATA-box, coding region, and 3′ box (the highest signal) of the *U1a* locus, but not in the downstream regions (U1-DS1 and U1-DS2) of the U1a 3′ box (Fig 3F and S3C Fig, S1 Data). These results show the occupancy of DSP1 at the snRNA loci, which, together with the fact that *dsp1-1* causes the defection of snRNA processing, demonstrates that DSP1 has a direct role in snRNA biogenesis. Both *U1a* and *U2*.*3* were transcribed through the DS1 region (S3D Fig). The absence of DSP1 in the DS1 region suggests that DSP1 may not travel through the 3′ box or be released at the 3′ box after cleavage.

### DSP1 Associates with Pol II in a DNA/RNA-Dependent Manner and Influences Its Occupancy in the *U2*.*3* and *U1a* Loci

The occupancy of DSP1 at the snRNA loci prompted us to test the interaction between DSP1 and Pol II by a co-immunoprecipitation (Co-IP) assay [34]. GFP-DSP1 and RPB2 (the second-largest subunit of Pol II) were able to reciprocally co-IP (Fig 3G and 3H). In contrast, GFP did not interact with RPB2 (Fig 3G and 3H). In addition, GFP-DSP1 and RPB2 proteins were not detected in the “no-antibody” reactions. These results confirm a DSP1-Pol II association. We further examined the dependence of the DSP1–Pol II interaction on DNAs/RNAs. Treatments with either DNase I or RNase A reduced the interaction of DSP1 with Pol II (Fig 3I), whereas micrococcal nuclease, which acts on both RNAs and DNAs, abolished the DSP1–Pol II interaction (Fig 3J).

We next evaluated the effect of *dsp1-1* on Pol II occupancy at the *U2*.*3* locus in a ChIP assay using anti-RPB2 antibodies. As expected, Pol II occupied the *U2*.*3* and *ACTIN2* loci, but not the Pol II C1 locus (an intergenic DNA fragment between At2g17470 and At2g17460) (Fig 3K and 3L) [35]. *dsp1-1* reduced Pol II occupancy at the USE, TATA box, and U2.3C of the *U2*.*3* locus, but not at the *ACTIN2* locus (Fig 3K and 3L, S1 Data). Interestingly, *dsp1-1* did not alter the occupancy of Pol II at the 3′ box (Fig 3K). *dsp1-1* had a similar effect of Pol II occupancy at various regions of the *U1a* locus (Fig 3M and S1 Data). These results suggest that DSP1 is required for proper occupancy of Pol II at snRNA loci.

### CPSF73-I Is Required for Pol II-Dependent snRNA Processing

**Fig 4:**
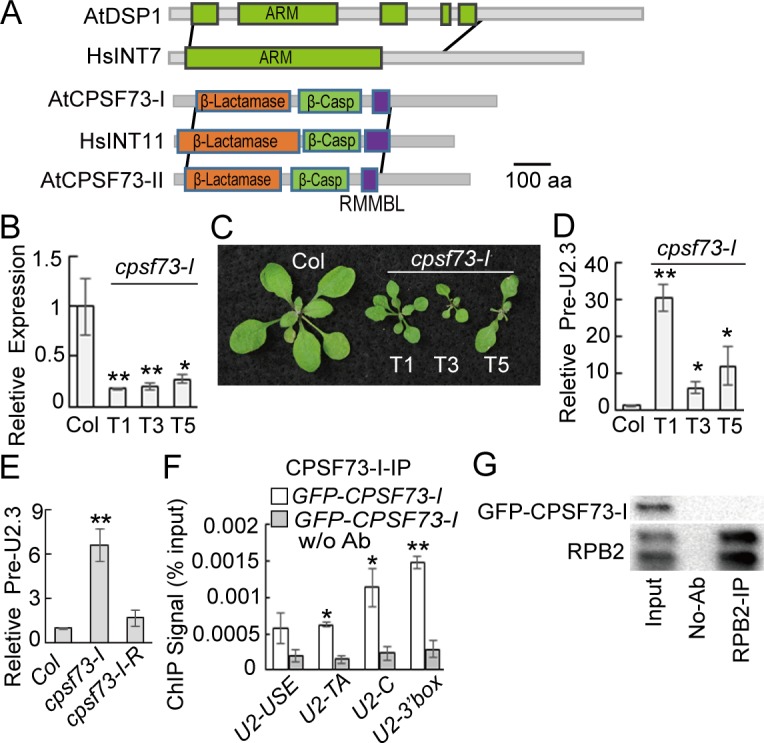
CPSF73-I is required for pre-snRNA processing. **(A)** Diagram showing protein alignments. The grey lines define the homologous regions of two proteins. Protein domains are indicated in color. ARM: armadillo-like fold; β-Casp: After metallo‐β‐lactamase‐associated CPSF Artemis SNM1/PSO2; RMMBL: RNA-metabolizing metallo-beta-lactamase. **(B–D)** The effect of *amiR*^*CPSF73-I*^ on plant development and the transcript levels of *CPSF73-I* and *pre-U2*.*3 snRNA*. *cpsf73-I*: *amiR*^*CPSF73-I*^. **(E)** Expression of an *amiR*^*CPSF73-*I^ resistance CPSF73-I transgene recovers the levels of pre-U2.3 in the *amiR*^*CPSF73-I*^ transgenic line. The levels of *pre-U2*.*3* snRNAs and *CPSF73-I* were normalized to those of *UBQ5* and compared with Col. Error bars indicate SD of three technical replicates (**p < 0.01). **(F)** The occupancy of CFPSF73-I at the U2.3 locus detected by ChIP. DNA co-purified with CPSF73-I was analyzed using qPCR. ChIP was performed on *N*. *benthamiana* leaves harboring the *GFP-CPSF73*-I and *pU2*::*pre-U2-GUS* transgenes. **(G)** CPSF73-I does not interact with Pol II. IP was performed using *Arabidopsis* harboring the *35S*::*GFP-CPSF73*-I transgene.

DSP1 does not contain any known nuclease domains, suggesting that it may associate with other proteins to act in snRNA maturation. DSP1 is a conserved protein in higher plants and contains an N-terminal armadillo (ARM)-like fold (Fig 4A and S4A Fig), which arranges in a regular right-handed super helix that provides a solvent-accessible surface for binding large substrates, such as proteins and nucleic acids, and a C-terminal region of unknown function [36]. We found that the ARM domain of DSP1 shared ~25% similarity with that of the integrator subunit 7 (INT7) of metazoans (Fig 4A). This led us to suspect that an INT-like complex might exist in plants. If so, an INT11 (the catalytic subunit of INT)-like nuclease should function in snRNA processing in plants. *Arabidopsis* encodes two INT11-like nucleases, CPSF73-I and CPSF73-II, which are conserved in higher plants (S4B Fig) [37,38]. Because they lack the characteristic C-terminal region of INT11 (Fig 4A), which is essential for snRNA maturation [39], and act as the catalytic components of the CPSF complex to cleave pre-mRNA 3′ end [37,38], CPSF73-I and CPSF73-II were never thought to act on snRNA processing. However, CPSF73-I is essential for both pollen and embryo development [38]. This resembles the effect of DSP1 on plant development, suggesting that CPSF73-I might be the nuclease that processes snRNAs in plants. To test this, we used an artificial miRNA (*amiR*^*CPSF73-I*^) to knockdown the expression of *CPSF73-I* (Fig 4B and S4C Fig, S1 Data) [40]. The reduced expression of *CPSF73-I* in the *amiR*^*CPSF73-I*^ lines caused developmental defects and increased the levels of *pre-U2*.*3* snRNAs (Fig 4C and 4D, S1 Data). Expression of an *amiR*^*CPSF73-I*^-resistant *CPSF73-I (CPSF73-I-R)* in the *amiR*^*CPSF73-I*^ lines recovered the levels of *pre-U2*.*3* snRNA (Fig 4E and S1 Data), suggesting that CPSF73-I is required for snRNA 3′ end maturation. Next, we tested if CPSF73-II also had a role in pre-snRNA processing (S4C Fig). Although *amiR*^*CPSF73-II*^ reduced the expression of *CPSF73-II*, resulting in pleiotropic developmental defects (S4C–S4E Fig and S1 Data), it did not affect the levels of *pre-snRNAs* (S4F Fig and S1 Data). We also examined whether CPSF100, which partners with CPSF73-I to process pre-mRNAs, is required for snRNA processing. However, a knockdown of CPSF100 by *amiR*^*CPSF100*^ did not alter the levels of pre-U2.3 RNAs (S4C, S4G and S4H Fig and S1 Data).

The above results suggest the presence of a CPSF73-I containing complex that acts on pre-snRNAs. Indeed, size-exclusion high performance liquid chromatography (HPLC) detected a ~670 kDa (eluted at 93–102 min) CPSF73-I-containing complex and a larger complex (eluted at 72–78 min) besides CPSF73-I monomers in the protein extracts of the transgenic plants harboring a *35S*::*GFP-CPSF73-I* transgene (S4I Fig). The ~670 kDa complex, but not the larger one, was able to process pre-U.2 snRNAs (S4J Fig). Next, we tested if this ~670 kDa complex could act on pre-mRNAs using a 5′ end [P^32^]-labeled RNA (RSB-3; ~380 nt) that covers the 3′-UTR of an rubisco small subunit gene (At5g38420) [41]. Proper 3′ end processing of RSB-3 would generate a ~240 nt RNA fragment and a ~190 nt RNA fragment due to the presence of two poly (A) sites (S4L Fig) [41]. The 670 kDa complex did not process RSB-3 (S4L Fig). We also examined the effect of *dsp1-1* on the formation of the CPSF73-I complex and its activity. The size of the snRNA-processing complex became smaller in *dsp1-1* relative to that in Col, and its pre-U2.3 snRNA processing activity was reduced (S4I and S4K Fig). In contrast, the larger complex was still intact in *dsp1-1* (S4D Fig).

We further examined the occupancy of CPSF73-I at the *U2*.*3* locus in *N*. *benthamiana* leaves harboring both GFP-CPSF73-I and *pU2*::*pre-U2-GUS*. ChIP assay detected the occupancy of CPSF73-I at the *pU2*::*U2*.*3-GUS* gene, with the highest occupancy at the 3′ box (Fig 4F and S1 Data). We also tested the interaction of CPSF73-I with Pol II in Col harboring a *35S*::*GFP-CPSF73-I* transgene. However, unlike DSP1, CPSF73-I did not interact with Pol II (Fig 4G).

### Identification of Other Components Required for snRNA Processing

**Fig 5:**
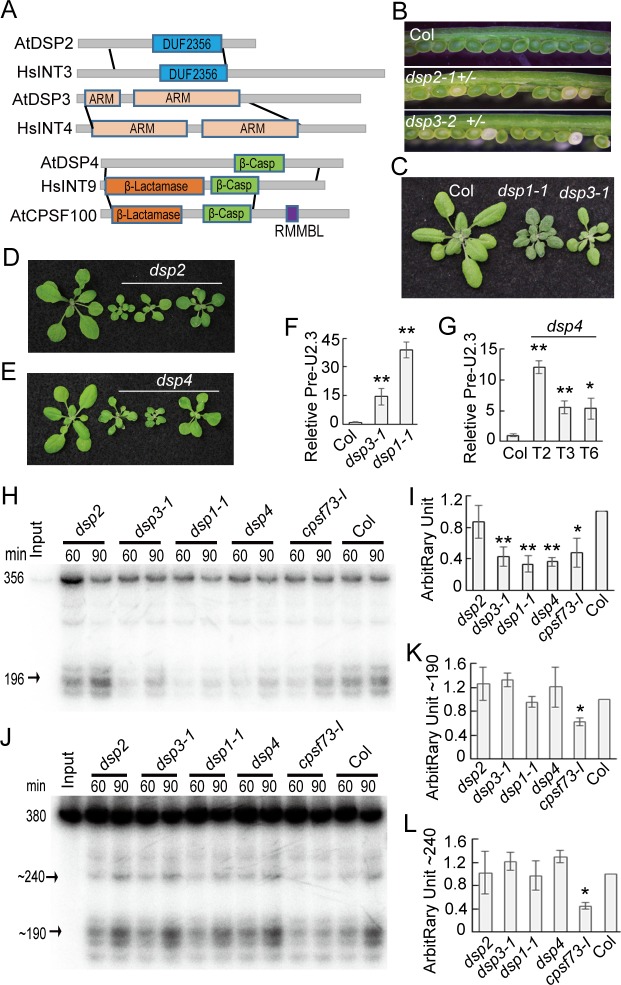
Identification of other components involved in snRNA maturation. **(A)** Schemes showing DSP2, DSP3, DSP4, and their human homologs. The grey lines define the homologous regions of two proteins. Protein domains are indicated in color. DUF2356: Domain of unknown function 2356; β-Casp: After metallo‐β‐lactamase‐associated CPSF Artemis SNM1/PSO2; RMMBL: RNA-metabolizing metallo-beta-lactamase. **(B)** Dissected green siliques of Col, *DSP2/dsp2*, and *DSP3*/*dsp3-2*. (**C–E)** Three-week-old seedlings of various genotypes. *dsp2*: *amiR*^*DSP2*^; *dsp4*: *amiR*^*DSP4*^. **(F)** and **(G)** The accumulation of *pre-U2*.*3* snRNAs in various genotypes. Transcript levels of *DSP2*, *DSP4*, and *pre-U2*.*3* snRNA in various mutants were normalized to *UBQ* and compared with those in Col (value set as 1). **p < 0.01, *p < 0.05 (*t* test). **(H)** In vitro processing of *pre-U2*.*3* snRNAs in nuclear protein extracts from various genotypes. **(I)** Quantification of mature *U2*.*3* snRNA production in various mutants relative to their levels in Col. *dsp2*: *amiR*^*DSP2*^ (T2); *dsp4*: *amiR*^*DSP4*^ (T4); *cpsf73-I*: *amiR*^*CPSF73-I*^ (T3). **(J)** In vitro processing of the 3′-UTR of the Rubisco small subunit gene (RSB-3) in nuclear protein extracts from various genotypes. **(K)** and **(L)** Quantification of ~190 and 240 nt cleavage products generated in various mutants relative to their levels in Col. *dsp2*: *amiR*^*DSP2*^ (T2); *dsp4*: *amiR*^*DSP4*^ (T4); *cpsf73-I*: *amiR*^*CPSF73-I*^ (T3). The radioactive signal of cleavage products from *U2*.*3* snRNAs or RSB-3 was quantified with Quantity One and then normalized to input. The levels of cleavage products generated by *dsp1-1*, *dsp2*, *dsp3-1*, *dsp4*, or *cpsf73-I* were then compared with those of Col, respectively. The value of Col was set to 1. The value represents mean of three repeats. **p < 0.01; *p < 0.05 (*t* test).

Next we sought to identify additional proteins acting in snRNA maturation by searching for *Arabidopsis* homologs of other INT subunits. We identified At4g14590 (named DSP2), At3g08800 (named DSP3; also known as SHORT-ROOT INTERACTING EMBRYONIC LETHAL, SIEL) [42], and At3g07530 (named DSP4) as potential homologs of INT3, INT4, and INT9, respectively (Fig 5A). Among them, DSP2 is approximately half size of INT3 and shares ~57% similarity with the N-terminal fragment (aa, 1–490) of INT3 (Fig 5A). The ARM domain, but not other regions, of DSP3 shared similarities with INT4 (Fig 5A). DSP4 has ~46% similarity with INT9 (Fig 5A). Like DSP1 and CPSF73-I, these proteins are conserved in higher plants (S5A–S5C Fig).

We evaluated if DSP2, DSP3, and DSP4 were required for snRNA processing using their loss-of-function mutants. The DNA knockout mutants for DSP2 (CS848944) and DSP3 (SALK_089544; *dsp3-2*; also known as *seil-2*) displayed embryo lethality (Fig 5B and S5D Fig), whereas expression of *DSP4* was not altered in the available T-DNA insertion mutants (SALK_005904; S5D–S5F Fig). We thus obtained a weak allele of *DSP3* (SALK_086160, *dsp3-1; siel-4*), in which a T-DNA insertion reduced the expression levels of *DSP3* and constructed knockdown lines of *DSP2 (amiR*^*DSP2*^) and *DSP4* (*amiR*^*DSP4*^) with artificial miRNAs (S5D–S5G Fig). *dsp3-1*, *amiR*^*DSP2*^,and *amiR*^*DSP4*^ reduced the expression *of DSP3*, *DSP2*, *and DSP4*, respectively, and caused pleiotropic developmental defects (Fig 5C–5E and S5F and S5H–S5I Fig, S1 Data). qRT-PCR showed that the levels of *pre-U2*.*3* snRNAs were increased in *dsp3-1* and *amiR*^*DSP4*^ relative to those in WT (Fig 5F and 5G, S1 Data), suggesting that they might act in snRNA processing. However, the levels of *pre-U2*.*3* snRNAs were not altered or slightly lower in *amiR*^*DSP2*^ relative to those in WT (S5J Fig and S1 Data), agreeing with a dispensable role of INT3 for pre-snRNA maturation [15].

### DSP1, DSP2, DSP3, and DSP4 Do Not Affect Pre-mRNA 3′ End Processing

To confirm the role of DSP2, DSP3, DSP4, and CPSF73-I in snRNA maturation, we examined their effect on the 3′ end cleavage of *pre-U2*.*3* snRNAs using the in vitro processing assay. The accumulation of mature snRNAs generated from *pre-U2*.*3* and *pre-U2*.*3-pG* was lower in nuclear protein extracts from *dsp3-1*, *amiR*^*DSP4*^, or *amiR*^*CPSF73-I*^ than from WT (Fig 5H and 5I and S6A Fig, S1 Data). In contrast, *amiR*^*DSP2*^ did not impair *pre-U2*.*3* and *pre-U2*.*3-pG* processing (Fig 5H and 5I and S6A Fig, S1 Data). In addition, the expression of *CPSF73-I-R* in the *amiR*^*CPSF73-I*^ line fully recovered the processing of *pre-U2*.*3-pG* snRNA (S6B Fig). These results demonstrate that, like DSP1 and CSPF73-I, DSP3 and DSP4 are required for snRNA processing.

The involvement of CPSF73-I in both pre-mRNA and pre-snRNA processing raised the possibility that the DSP proteins might also function in pre-mRNA 3′ end cleavage. Therefore, we tested their effect on the 3′ end processing of the *RSB-3* RNA (S4L Fig) using nuclear protein extracts. As expected, the 3′ end processing efficiency of *RSB-3* was reduced in *amiR*^*CPSF73-I*^ and *amiR*^*CPSF73-II*^ relative to WT at various time points (Fig 5J–5L and S6C Fig, S1 Data). In contrast, RSB-3 processing in protein extracts from *dsp1-1*, *dsp3-1*, *amiR*^*DSP2*^, and *amiR*^*DSP4*^ was comparable with that of WT (Fig 5J–5L and S1 Data), suggesting that DSP1, DSP2, DSP3, and DSP4 are not required for pre-mRNA 3′ end processing. To further validate the result, we monitored the 3′ end formation of *FCA* mRNA, which is known to be affected by the pre-mRNA 3′ end processing complex [43], in *dsp*, *amiR*^*CPSF73-I*^, and *amiR*^*CPSF73-II*^ by northern blot. *amiR*^*CPSF73-I*^ and *amiR*^*CPSF73-II*^, but not *dsp1-1*, *dsp3-1*, *amiR*^*DSP2*^, and *amiR*^*DSP4*^, altered the 3′ end formation of *FCA* (S6D Fig).

### DSP1, DSP2, DSP3, DSP4, and CPSF73-I Form a Complex

**Fig 6:**
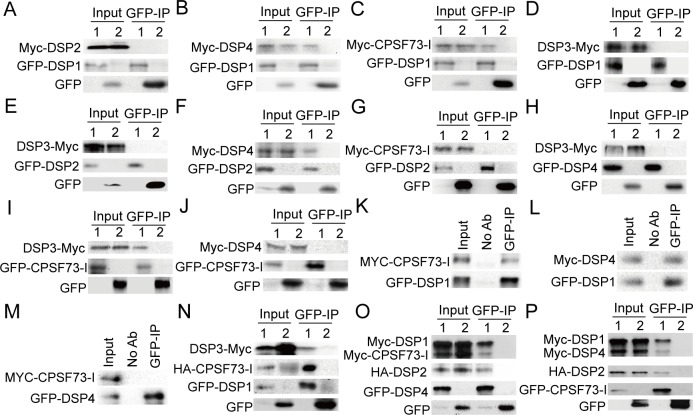
DSP1, DSP2, DSP3, DSP4, and CPSF73-I form a complex. **(A–D)** The interactions of DSP1 with DSP2, DSP3, DSP4, or CPSF73-I. **(E–G)** The interactions of DSP2 with DSP3, DSP4, or CPSF73-I. **(H)** The interaction of DSP4 with DSP3. **(I)** and **(J)** The interactions of CPSF73-I with DSP3-MYC or MYC-DSP4. GFP tagged proteins (1) or GFP (2) were co-expressed with various proteins as indicated on the left of the top row in *N*. *benthamiana*, except for DSP3-MYC. In vitro–translated DSP3-MYC was added to protein extracts containing GFP-tagged proteins (1) or GFP (2) as indicated on the left of the top row. Proteins detected by western blot are indicated on the left. **(K)** and **(L)** The interactions of DSP1 with CPSF73-I or DSP4 in *Arabidopsis*. **(M)** The interactions of DSP4 with CPSF73-I in *Arabidopsis*. IP was performed on protein extracts from *Arabidopsis* harboring GFP-DSP1/MYC-CPSF73-I, GFP-DSP1/MYC-DSP4, or GFP-DSP4/MYC-CPSF73-I with anti-GFP antibodies. Proteins detected by western blot are indicated on the left. **(N)** DSP1 pulls down DSP3 in the presence of CPSF73-I. GFP-DSP1 (1) or GFP (2) were co-expressed with HA-CPSF73-I. Protein extracts were mixed with in vitro–translated DSP3-MYC. Proteins detected by western blot are indicated on the left. **(O)** DSP4 pulls down DSP1, DSP2, and CPSF73-I. GFP-DSP4 (1) or GFP (2) were co-expressed with MYC-DSP1, HA-DSP2, and MYC-CPSF73-I in *N*. *benthamiana*. Proteins detected by western blot are indicated on the left. **(P)** CPSF73-I pulls down DSP1, DSP2, and DSP4. GFP-CPSF73-I (1) or GFP (2) were co-expressed with MYC-DSP1, HA-DSP2, and MYC.

The involvement of DSP1, DSP3, DSP4, and CPSF73-I in the snRNA maturation raised the possibility that they may form a complex to cleave pre-snRNAs. To test this possibility, we first examined the interaction of DSP1 with the other proteins using co-IP. DSP2 was included in this experiment because its homolog INT3 is a component of the INT complex [14]. We also included CPSF100 as a control because it is a homolog of DSP4 but does not affect snRNA processing. *GFP-DSP1* was transiently co-expressed with *MYC-DSP2*, *MYC-CPSF100*, *MYC-DSP4*, or *MYC-CPSF73-I* in *N*. *benthamiana* as described previously [34]. MYC-DSP4 and MYC-CPSF73I, but not MYC-DSP2 and MYC-CPSF100, were detected in the GPF-DSP1 precipitates (Fig 6A–6C and S7A Fig). In addition, the control, GFP, did not co-IP with MYC-DSP2, MYC-DSP4, and MYC-CPSF73-I (Fig 6A–6C). We were unable to express the recombinant DSP3 protein in either *N*. *benthamiana* or *Escherichia coli*, likely because it is extremely unstable. To test the interaction of DSP1 with DSP3, we generated a recombinant DSP3-MYC protein using an in vitro translation system as described [44]. However, DSP1 did not co-IP with DSP3-MYC (Fig 6D). These results support the interaction of DSP1 with DSP4 and CPSF73-I, but not with DSP2, DSP3, and CPSF100. We further tested the interaction of GFP-DSP2 with DSP3-MYC, MYC-DSP4, or MYC-CPSF73-I. GFP-DSP2 interacted with MYC-DSP4 but not with DSP3-MYC and MYC-CPSF73-I (Fig 6E–6G). Co-IP/pull down assays also showed that DSP4 did not interact with DSP3 and CPSF73-I, but DSP3 did interact with CPSF73-I (Fig 6H–6J). To confirm these protein interactions, we performed a bimolecular fluorescence complementation (BiFC) assay (S1 Text) [45]. In this assay, the paired proteins, which were fused to the N-terminal fragment of yellow fluorescent protein (nYFP) or to the C-terminal fragment of YFP (cYFP), respectively, were introduced into tobacco cells by infiltration. The interaction of the two protein partners will result in a functional YFP [34]. As expected, the DSP1–DSP4, DSP1–CPSF73-I, DSP2–DSP4 interactions, but not the DSP1–DSP2, DSP2–CPSF73-I, and DSP4–CPSF73-I interactions, were confirmed (S7B Fig). We further validated the protein interactions using stable transgenic lines harboring GFP-*DSP1*/*MYC-CPSF73-I*, *GFP-DSP1*/*MYC-DSP4*, or *GFP-DSP4*/*MYC-CPSF73-I* transgenes. As observed in tobacco, we detected the DSP1–DSP4 and DSP1–CPSF73-I interactions, but not the DSP4–CPSF73-I interaction, in *Arabidopsis* (Fig 6K–6M).

**Fig 7:**
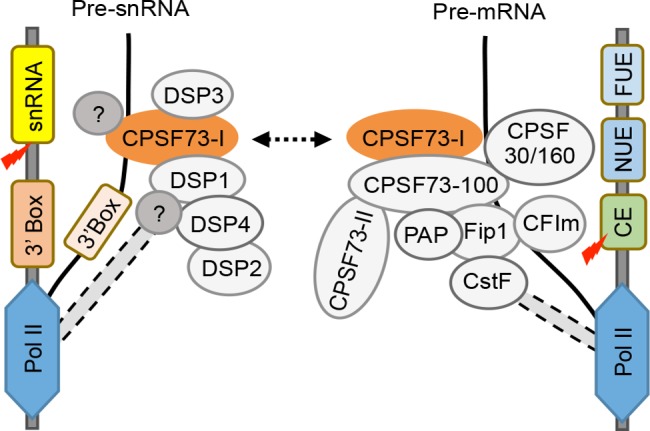
Models for the 3′ end processing of snRNAs and mRNAs in *Arabidopsis*. DSP1, DSP2, DSP3, DSP4, and CPSF73-I form a complex to co-transcriptionally cleave the pre-snRNAs upstream of the 3′ box. This complex may contain additional proteins. In contrast, a complex composed of CPSF100, CPSF73-I, and other proteins acts specifically on the 3′ end of pre-mRNAs. However, whether these two complexes have overlapping functions on some substrates remains to be investigated. FUE: far-upstream element; NUE: near-upstream element; CE: Cleavage element; PAP: Poly (A) polymerase; Fip1: Factor interacting with poly (A) polymerase 1; CstF: Cleavage stimulatory factor; CFIm: Mammalian cleavage factor I. The dashed lines indicate potential physical interaction. The dashed arrow indicates potential connection between the DSP1 complex and the CPSF complex.

Next, we asked if these proteins could co-exist in a complex. We found that GFP-DSP1 pulled down both CPSF73-I and DSP3 from protein extracts containing DSP3-MYC, GFP-DSP1, and HA-CPSF73-I (Fig 6N). In addition, when co-expressed, DSP4 co-IPed with DSP1, DSP2, and CPSF73-I, while CPSF73-I co-IPed with DSP1, DSP2, and DSP4 (Fig 6O and 6P). These results demonstrate that DSP1, DSP2, DSP3, DSP4, and CPSF73-I likely form a complex to process snRNAs (Fig 7).

## Discussion

We identified a conserved complex essential for 3′ end maturation of Pol II-dependent snRNAs in plants. This complex contains at least five proteins, including DSP1, DSP2, DSP3, DSP4, and CPSF73-I. In this complex, DSP1 bridges DSP4 and CPSF73-I, whereas DSP2 and DSP3 may act as accessory components of DSP4 and CPSF73-I, respectively (Fig 7). More importantly, we show that CPSF73-I likely is the catalytic component for snRNA 3′ end processing. This result shows that higher plants use the same enzyme to process both pre-mRNAs and pre-snRNAs. However, the two CPSF73-containing complexes might function separately in snRNA and pre-mRNA maturation (Fig 7), as the *dsp* mutations do not impair the mRNA 3′ end processing and a knockdown of CPSF-100 or CPSF73-II does not affect snRNA 3′ end maturation. Furthermore, mass spectrometry analyses did not identify any DSP proteins in the CPSF-100 complex [46]. Consistent with this, DSP1 interacts with DSP4 but not its homolog CPSF-100. In contrast to what we have discovered in plants, in metazoans, CPSF73 and its paralog, INT11, are used to process pre-mRNAs and pre-snRNAs, respectively. However, the similarities of some DSP proteins with their counterparts in INT raise the possibilities that a common ancestor complex containing CPSF73 might have been used to process pre-snRNAs before divergence between metazoans and plants and that CPSF73 may be subject to sub functionalization in metazoans.

How does the DSP1 complex recognize and process pre-snRNAs? The occupancy of DSP1 and CPSF73-I at snRNA loci and the DSP1-Pol II association support the idea that the DSP1 complex processes pre-snRNAs co-transcriptionally. Both CPSF73 and DSP1 have the highest occupancy at the 3′ box, and mutations in the 3′ box greatly reduced the activity of the DSP1 complex, demonstrating that the 3′ box is essential for the DSP1 complex to recognize the cleavage site. In metazoans, Pol II plays key roles in recruiting INT to snRNA loci and transcription initiation is essential for snRNA processing [6,17–21]. However, in plants, blocking transcription initiation only has a minor effect on snRNA processing [31]. In addition, DSP1 interacts with Pol II in a DNA/RNA-dependent manner, whereas CPSF73-I and DSP4 do not associate with Pol II (Fig 4G and S5K Fig). These results suggest that Pol II is not crucial for recruiting the DSP1-CPSF73 complex to the snRNA loci, although we cannot completely rule out this possibility. Perhaps the DSP1 complex can recognize specific sequence in the promoters of snRNAs. Alternatively, the DSP1 complex might be recruited to snRNA loci through its interaction with some snRNA-specific transcription factors. Clearly, all these possibilities need to be examined in the near future.

The DSP1 complex may have other roles in snRNA biogenesis. The facts that the DSP1 interacts with the snRNA promoters and that the *dsp1-1* mutation reduced the occupancy of Pol II at the promoters and coding regions of U1 and U2 snRNA genes support that the DSP complex promotes the transcription of Pol-II dependent snRNAs. In further support of this, the transcript levels of *preU2m-GUS* RNAs are slightly lower in *dsp1-1* than in WT (Fig 2H). However, it is not clear whether the DSP1 complex directly or indirectly regulates snRNA transcription. The DSP complex may also positively contribute to Pol II releasing at the snRNA 3′ end, because the 3′ end cleavage will help transcription termination. If so, the Pol II occupancy at the 3′ end of snRNA loci should be increased in *dsp1-1*. However, we observed unchanged Pol II occupancy at the 3′ end in *dsp1-1* relative to Col. This result likely reflects the combined effects of DSP1 on snRNA transcription and 3′ end processing.

Besides snRNA biogenesis, the DSP complex may have other functions, given the facts that lack of DSP2, which has a minor role in snRNA processing, causes embryo lethality and developmental defects (Fig 5) and that *dsp1-1*, in which the abundance of mature snRNAs is comparable to that of WT, still displays pleiotropic developmental defects (Figs 1 and 2). In fact, DSP3 (known as SIEL) has been shown to promote root patterning through interacting with SHR, a transcription factor, and promoting its movement [42]. It will be interesting to test whether other DSP components have similar functions in root patterning. In metazoans, INT not only functions in snRNA processing, but also controls the transcription termination of some mRNAs, the biogenesis of enhancer RNAs, which are noncoding RNAs regulating gene expression, and the biogenesis of some viral-derived miRNAs [33,47–50]. It is possible that the DSP complex plays similar roles in plants. We identified several mRNAs containing the 3′ box at their 3′ end from the *Arabidopsis* genome. However, the DSP complex does not affect their processing. Thus, it remains to be determined if the DSP complex has other substrates and, if so, what these substrates are.

## Experimental Procedure

### Plant Materials

T-DNA insertion mutants including CS848944, SALK_089544, SALK_005904, SALK_036641, CS16199, and SALK_086160 were obtained from the *Arabidopsis* stock center (www.arabidopsis.org); all are in the Col genetic background. Transgenic lines (Col background) harboring *pU2*::*pre-U2-GUS* or *pU2*::*pre-U2m-GUS* were crossed to *dsp1-1*. In the F2 population, *DSP1*^*+*^ (*DSP1/DSP1*; *DSP1/dsp1-1*) plants and *dsp1-1* containing the transgenes were identified by genotyping of T-DNA and GUS using primers listed in S2 Table.

### Complementation Assay

A 6.4 kb genomic fragment containing the *DSP1* promoter and coding regions was PCR amplified, cloned into pENTR/SD/D-TOPO, and subsequently cloned into the binary vector pGWB4. The resulting plasmid was transformed into *dsp1-1*, and transgenic plants were screened for Hygromycin resistance.

### Plasmids Construction

*DSP1* cDNA was amplified by RT-PCR, cloned into pENTR/SD/D-TOPO, and subsequently cloned into pEG104 [51] to generate the *35S*::*GFP-DSP1* fusion vector. A genomic fragment containing the *U2*.*3* gene promoter, snRNA coding region, and 3′ box region was PCR amplified and cloned into pMDC164 to generate *pU2*::*pre-U2-GUS*. The 3′ box of *pre-U2-GUS* was then mutated to generate *pU2*::*pre-U2m-GUS* using a Site-Directed Mutagenesis Kit (Stratagene). The primers used for plasmid construction are listed in S2 Table.

### Microscopy

Siliques of different developmental stages were dissected with hypodermic needles, mounted on microscope slides in a clearing agent (Visikol) overnight, and then observed with a confocal microscope. To visualize GUS expression, samples were immersed in the GUS staining solution for 12 h in the dark. The stained samples were treated with 70% ethanol to remove chlorophyll before observation using a dissecting microscope.

### RT-PCR Analysis

cDNA was synthesized from 2 μg of total RNA with reverse transcriptase (Invitrogen) and random primers. qPCR was performed in triplicate on a Bio-Rad IQcycler apparatus with the Quantitech SYBR green kit (Bio-Rad). The primers used for PCR are listed in S2 Table.

### In Vitro Processing Assay of RNAs

In vitro processing assays of pre-U2.3 and the 3′ UTR of a Rubisco small subunit gene (RSB-3) were performed as described [13,41]. Briefly, DNA templates used for in vitro transcription of pre-U2.3 and RBS-3 were amplified using T7 promoter-anchored primers (S2 Table). A 5′ end [^32^P]-labeled *pre-U2*.*3* snRNA was incubated with 2 μg nuclear proteins in a 20 μl reaction, while [^32^P]-labeled RSB-3 was cleaved by 4 μg nuclear proteins in a 20 μl reaction. After reactions were stopped at various time points, RNAs were extracted, purified, and resolved on a PAGE gel. Radioactive signals were detected by PhosphorImager and quantified by Quantity One.

### ChIP Assay

ChIPs with anti-GFP and anti-Pol II were performed as described [34]. Anti-RPB2 (Abcam) and anti-GFP antibodies (Clontech) were used for IP. Enrichment of DNA fragments was measured by qPCR. The primers used in ChIP-PCR are listed in S2 Table.

### Protein–Protein Interaction Assay

To test DSP1–PoII interaction, proteins were extracted from *dsp1-1* harboring the *GFP-DSP1* transgene. To test CPSF73-I–Pol II interactions, proteins were extracted from *N*. *benthamiana* transiently expressing *GFP-CPSF73-I*. To test the interactions among DSP1, DSP2, DSP4, and CPSF73-I, proteins were co-expressed in *N*. *benthamiana*. To analyze multi-protein–containing complexes, samples were treated with formaldehyde to fix protein–protein interactions as described [52]. To test the interaction of DSP3 with other proteins, a *DSP3-MYC* fragment was generated using primers containing elements required for in vitro transcription and translation (S2 Table). The resulting DNA fragment was used as a template to synthesize DSP3-MYC protein using a PURExpress In Vitro Protein Synthesis Kit (New England Biolabs). To obtain plants harboring two transgenes, transgenic *Arabidopsis* harboring *GFP-DSP1* was crossed with transgenic plants containing MYC-CPSF73-I or MYC-DSP4 transgenic, whereas transgenic *Arabidopsis* harboring GFP-DSP4 was crossed with MYC-CPSF73-I transgenic lines. F1 plants harboring both transgenes were used for IP assay.

### Pollen Viability and Pollen Growth Assays

Pollen viability was examined after Alexander’s staining [53]. In vitro pollen growth assays were performed as described [54]. To examine pollen tube growth in Col-0 and *dsp1* in vivo, pistils were pollinated and collected 12 h later, then cleared and stained with decolorized aniline blue [54].

## Supporting Information

S1 Data Data underlying Fig 1, Fig 2, Fig 3, Fig 4, Fig 5, S1 Fig, S4 Fig and S5 Fig. (XLSX)

S1 Fig DSP1 is required for plant development and snRNA processing. **Related to Fig 1. (A)** A diagram showing the T-DNA insertion positions in the *DSP1* gene. Gray box: coding-region; Solid black line: intron; Gray arrowheads: primers used for T-DNA genotyping; Black arrowheads: primers used for RT-PCR analysis. **(B)** PCR analyses of DNAs isolated from Col-0 (WT), *dsp1-1*, and *DSP1*/*dsp1-2*. The primer combinations LP/RP and LBa1 (LB2)/RP are diagnostic for *DSP1*, and the T-DNA flanking genomic DNA, respectively. **(C)** RT-PCR analysis of the *DSP1* transcripts in *dsp1-1* and Col-0. Amplification of *UBIQUITIN 5* (*UBQ5*) was used as control. **(D)** Morphological phenotypes of Col and *dsp1-1*. **(E)** Palisade cells from Col and *dsp1-1*. **(F)** The siliques from three genotypes. **(G)** The accumulation of *pre-U2*.*3* snRNAs detected by qRT-PCR in indicated genotypes. The levels of *pre-U2*.*3* snRNAs were normalized to those of *UBQ5* and compared with Col. Error bars indicate SD of three technical replications (**p < 0.01). Three biological replicates showed similar results. **(H)** Arrest of embryo development. Siliques with most embryos at the torpedo stage from heterozygous *DSP1*/*dsp1-2* plants were dissected. White arrow indicates the embryos arrested at the globular stage. **(I)** Abnormal embryos observed in *dsp1-1*. **(J)** Pollens germinated in vitro. Images of pollen tubes were obtained at 12 h after germination. **(K)** In vivo pollens growth. Pistils were collected, cleared, stained with Aniline Blue, and visualized with light microscopy 12 h after pollination.
(TIF)

S2 Fig DSP1 is required for the snRNA 3′ end maturation. **Related to** **Fig** 2**. (A)** The abundance of various pre-snRNAs detected by RT-PCR. UBQ5 was amplified as a loading control. **(B)** Sequencing analyses of mature *U2*.*3* snRNAs in *dsp1-1*. Three forms of mature U2.3 snRNAs were identified. Colored arrows indicate the 3′ terminal nucleotide of *U2*.*3* snRNAs. The adapter sequence is covered with a blue box. **(C)** Diagram of snRNA probes used for RNase protection assay (RPA). The structure of pre-snRNA is shown on top. White and gray arrows represent primary and mature transcripts, respectively. Black arrow indicates antisense RPA probe. Grey and black lines indicate the protected fragments from RNase treatment. **(D)** The premature and mature *U1a* and *U2*.*3* snRNAs detected by RPA. Ten micrograms of total RNA were incubated with [^32^P]-labeled RNA probe and treated by RNase T1 and RNase A. After reactions, RNAs were separated on a PAGE gel and detected with a PhosphorImager. Black arrow indicates pre-snRNAs. Grey arrow indicates mature snRNAs. **(E)** The transcripts of three protein-coding genes detected by RT-PCR. *UBQ5* was amplified as a loading control. **(F)** In vitro processing of *pre-U2*, *pre-U2m*, *pre-U2-pG*, *and pre-U2-pA* RNAs. In vitro transcribed RNAs were [^32^P] labeled at 5′ end and processed in the nuclear protein extracts from Col for various times. *pre-U2-pG*: an 18-nt poly-G tail was added at the 3′ end of *pre-U2*.*3* snRNA. *pre-U2*.*3-pA*: an 18-nt poly-A tail was added at the 3′ end of *pre-U2*.*3* snRNA. After reaction, RNAs were resolved on PAGE gel and detected with a PhosphorImager. 1: reaction stopped at 0 min; 2: reaction stopped at 30 min; 3: reaction stopped at 60 min. **(G)** Sequence of the 3′ box and mutated 3′ box of the *U2*.*3* gene. 3′ box is defined by the red rectangle. Green letter: nucleotide that was changed. Red letter: mutated nucleotide. The colored arrow indicates possible cleavage position. **(H)** GUS activities in Col harboring *pU2*::*pre-U2-GUS*, or *pU2*::*pre-U2m-GUS*. **(I)** The expression levels of *pre-U2-GUS* RNAs and *pre-U2m-GUS* RNAs in *DSP*^*+*^ and *dsp1-1* detected by RT-PCR. Black arrows: primers used for PCR. *UBQ5* was used as a loading control.
(TIF)

S3 Fig DSP1 binds the *U2*.*3* and *U1a* loci and affects the occupancy of Pol II at the snRNA loci. **Related to** **Fig** 3**. (A–C)** The occupancy DSP1 at the *U2*.*3*, *U1a*, and *ACTIN2* loci detected by ChIP in transgenic plants harboring *DSP1-GFP* or *GFP*. PCR was used to analyze DNAs co-purified with DSP1-GPF or GFP. **(D)** Detection of the 3′ end transcript of *U1a* and *U2*.*3* snRNAs in Col and *dsp1-1* by RT-PCR. DS1 and DS2 localize downstream of the 3′ box of the *U1a* or *U2*.*3* genes. PCR amplification of genomic DNA serves as a positive control.
(TIF)

S4 Fig Analyses of CPSF73-I, CPSF73-II, and CSPF100. **Related to** **Fig** 4**. (A)** and **(B)** Phylogenetic analyses of DSP1 and CPSF73 homologs in plants. The full-length protein sequences were used to construct a Maximum Likelihood tree based on the Jones–Taylor–Thornton model. Scale bar represent the estimated number of substitution per site. **(C)** Schemes of artificial miRNAs targeting *CPSF73-I*, *CPSF73-II*, and *CPSF100*. The degree of pairing between the amiRNA and target gene is shown. The red letters indicate the mutated nucleotides in amiRNA target region of CPSF73-I. Solid line represents Watson–Crick pairing and a “0” indicates a G-U pairing. (**D–F)** The effect of *amiR*^*CPSF73-II*^ on plant development and the transcript levels of *CPSF73-II* and *pre-U2*.*3 snRNA*. *cpsf73-II*: *amiR*^*CPSF73-II*^. The transcript levels of *CPSF73-II or pre-U2*.*3* in *amiRNA* lines were detected by qRT-PCR, normalized to *UBQ5* and compared with those in Col (value set as 1). **p < 0.01, *p < 0.05 (*t* test). (**G)** The transcript levels of *CPSF100* in three *amiR*^*CPSF100*^ lines detected by qRT-PCR. *cpsf100*: *amiR*^*CPSF100*^. *p < 0.05 (*t* test). **(H)** The transcript levels of *pre-U2*.*3* RNAs in three *amiR*^*CPSF100*^ lines detected by qRT-PCR. *cpsf100*: *amiR*^*CPSF100*^. **(I)** Gel filtration analysis of the CPSF73-I complex in Col and *dsp1-1*. Protein extracts from Col and *dsp1-1* harboring *GFP-CPSF73-I* were separated by HPLC. Eluted fractions were separated by SDS–PAGE and detected by western blotting. Elution times are shown on the top of the picture. **(J)** In vitro processing of *pre-U2*.*3* snRNAs by eluted proteins from Col. Elution times are shown on the top of picture. Reactions were stopped at 60 minutes. Arrow indicates mature snRNAs. **(K)** *dsp1-1* reduced the snRNA processing activity of the CPSF73-I complex. **(L)** In vitro processing of *RSB-3* using the CPSF73-I complex that acts on pre-snRNAs. Reaction using nuclear extracts from Col was used as positive control. RSB-3 represents the 3′ UTR of the Rubisco small subunit gene.
(TIF)

S5 Fig Analyses of the *dsp2*, *dsp3*, and *dsp4* mutants. **Related to** **Fig** 5. **(A–C)** Phylogenetic analysis of DSP2, DSP3, and DSP4 in plants. The full-length protein sequences were used to construct a Maximum Likelihood tree based on the Jones–Taylor–Thornton model. Scale bar represents the estimated number of substitution per site. **(D)** Schemes showing the T-DNA insertion positions in DSP2, DSP3, and DSP4. **(E)** PCR analyses of T-DNA insertion in various genotypes. The primer combinations LP/RP and LBa1 (LB2)/RP are diagnostic for genes and the T-DNA flanking genomic DNA, respectively. **(F)** RT-PCR analysis of the *DSP3* and *DSP4* transcripts in their T-DNA insertion mutants. *UBQ5* was amplified as a control. **(G)** Diagrams showing the artificial miRNAs targeting *DSP2* or *DSP4*. Solid line indicates Watson–Crick pairing and a “0” defines a G-U pairing. **(H)** and **(I)** Transcript levels of *DSP2* and *DSP4* in their amiRNA lines. *dsp2*: *amiR*^*DSP2*^; *dsp4*: *amiR*^*DSP4*^. The abundance of *DSP2* or *DSP4* in the amiRNA lines was normalized to *UBQ5* and compared with those in Col (set as 1). **p < 0.01, *p < 0.05 (*t* test). **(J)** The accumulation of *pre-U2*.*3* snRNAs in *amiR*^*DSP2*^. *dsp2*: *amiR*^*DSP2*^. Transcript levels of *pre-U2*.*3* snRNA were normalized to *UBQ* and compared with those in Col (value set as 1). **p < 0.01, *p < 0.05 (*t* test). **(K)** DSP4 does not interact with Pol II. IP was performed using *Arabidopsis* harboring the *GFP-DSP4* transgene
(TIF)

S6 Fig The processing of pre-snRNAs and *pre-*mRNA by the DSP complex. **Related to** **Fig** 5. **(A)** and **(B)** In vitro processing of *pre-U2*.*3-pG* snRNAs in the nuclear protein extracts from various genotypes. In vitro transcribed *pre-U2*.*3-pG* (*pre-U2*.*3* snRNAs with poly G at 3′ end) were [^32^P] labeled at 5′ end and processed in the nuclear protein extracts from various genotypes. *dsp2*: *amiR*^*DSP2*^*; dsp4*: *amiR*^*DSP4*^; *cpsf73-I*: *amiR*^*CPSF73-I*^**. (C)** In vitro processing of *RSB-3* in nuclear protein extracts from Col and *cpsf73-II*. *cpsf73-II*: *amiR*^*CPSF73-II*^. RSB-3: The 3′ UTR of the Rubisco small subunit gene. **(D)** The accumulation of *FCA* transcripts in various genotypes. FCA transcripts were detected by northern Blot. *UBQ5* was probed as loading control.
(TIF)

S7 Fig The interactions among DSP1, DSP2, DSP3, DSP4, and CPSF73-I. **Related to** **Fig** 6**. (A)** DSP1 does not interact with CPSF100. *GFP-DSP1* (1) or *GFP* (2) was co-expressed with *MYC-CPSF100* in *N*. *benthamiana*. Proteins detected by western blot were labeled on the left side of the picture. **(B)** BiFC analysis of the interactions among DSP1, DSP2, DSP4, and CPSF73-I. Respective pairs of cYFP and nYFP fused proteins were co-expressed in *N*. *benthamiana* leaves. Yellow fluorescence (green in image) signals were detected by confocal microscopy. Red fluorescence was from chlorophyll. Approximately 20 nuclei were examined for each pair, and an image is shown.
(TIF)

S1 Table Related to Fig 1. Genetic assay of male transmission in *dsp1-2* by reciprocal.
(DOCX)

S2 Table Primers used in this study (DOCX)

S1 Text Supplemental Experimental Procedures. (DOCX)

## Abbreviations

ARM: N-terminal armadillo
BiFC: bimolecular fluorescence complementation
ChIP: chromatin immunoprecipitation
Co-IP: co-immunoprecipitation
Col: Columbia-0
CPSF73: cleavage and polyadenylation specificity factor 73 kDa
CTD: C-terminal domain
cYFP: C-terminal fragment of YFP
DSE: distal sequence element
DSP1: defective in snRNA processing 1
dsp1-1: defective in snRNA processing 1–1
GFP: Green Fluorescent Protein
HPLC: High performance liquid chromatography
INT: integrator complex
INT7: integrator subunit 7
nt: nucleotide
nYFP: N-terminal fragment of YFP
Pol II: RNA polymerase II
pre-mRNAs: pre-messenger RNAs
pre-snRNAs: snRNA primary transcripts
PSE: proximal sequence element
qPCR: quantitative PCR
qRT-PCR: quantitative RT-PCR
RT-PCR: Reverse transcription PCR
SD: standard deviation
snRNAs: small nuclear RNAs
UBQ5: UBIQUITIN 5
USE: upstream sequence element
WT: wild type
YFP: Yellow Fluorescent Protein
