# Supplementary figures and images for: snRNA 3′ End Processing by a CPSF73-Containing Complex Essential for Development in Arabidopsis
Source: PLoS Biol. 2016 Oct 25;14(10):e1002571. doi: 10.1371/journal.pbio.1002571 (PMC5079582; doi:10.1371/journal.pbio.1002571)

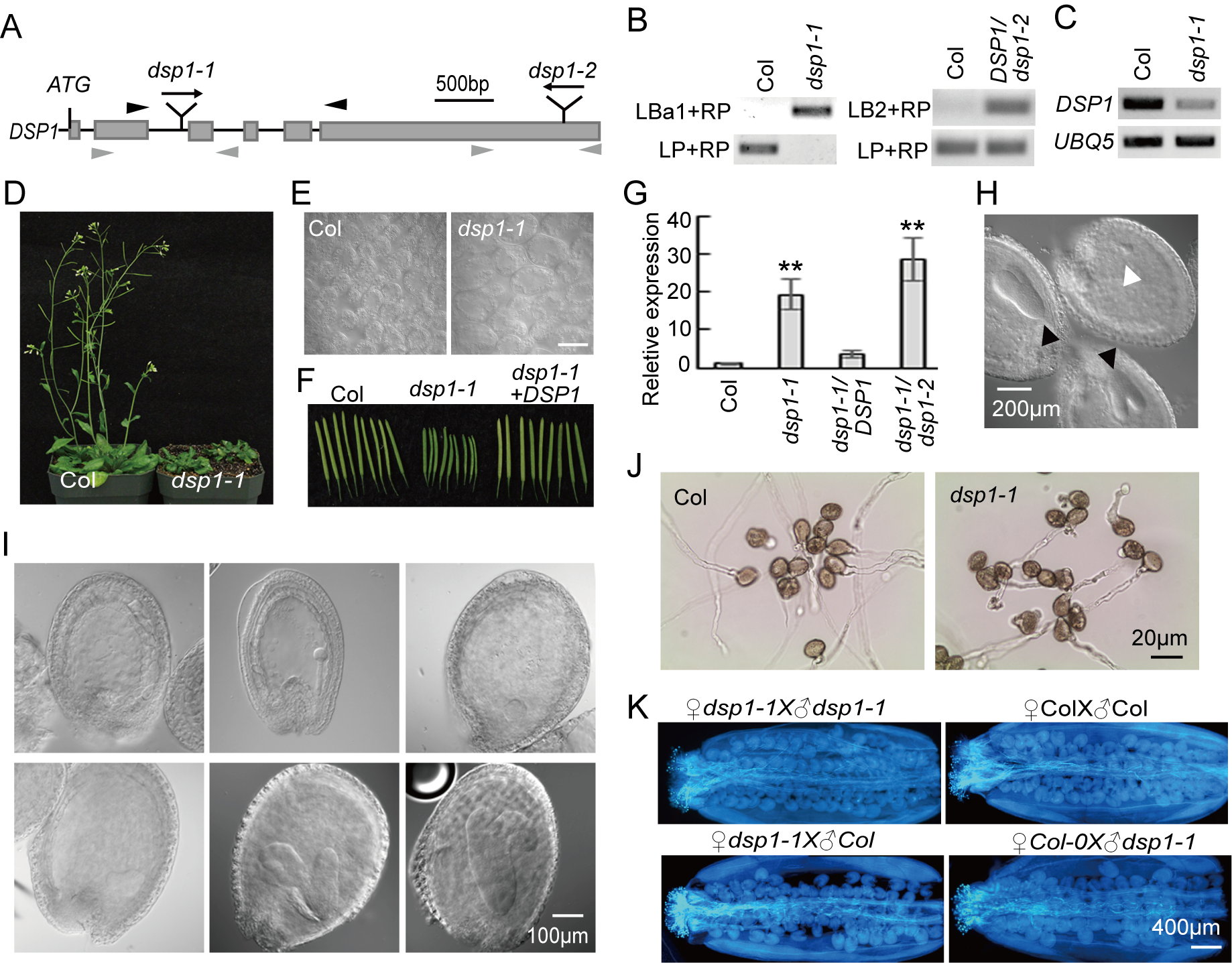

Supplement: S1 Fig — Related to Fig 1. (A) A diagram showing the T-DNA insertion positions in the DSP1 gene. Gray box: coding-region; Solid black line: intron; Gray arrowheads: primers used for T-DNA genotyping; Black arrowheads: primers used for RT-PCR analysis. (B) PCR analyses of DNAs isolated from Col-0 (WT), dsp1-1, and DSP1/dsp1-2. The primer combinations LP/RP and LBa1 (LB2)/RP are diagnostic for DSP1, and the T-DNA flanking genomic DNA, respectively. (C) RT-PCR analysis of the DSP1 transcripts in dsp1-1 and Col-0. Amplification of UBIQUITIN 5 (UBQ5) was used as control. (D) Morphological phenotypes of Col and dsp1-1. (E) Palisade cells from Col and dsp1-1. (F) The siliques from three genotypes. (G) The accumulation of pre-U2.3 snRNAs detected by qRT-PCR in indicated genotypes. The levels of pre-U2.3 snRNAs were normalized to those of UBQ5 and compared with Col. Error bars indicate SD of three technical replications (**p < 0.01). Three biological replicates showed similar results. (H) Arrest of embryo development. Siliques with most embryos at the torpedo stage from heterozygous DSP1/dsp1-2 plants were dissected. White arrow indicates the embryos arrested at the globular stage. (I) Abnormal embryos observed in dsp1-1. (J) Pollens germinated in vitro. Images of pollen tubes were obtained at 12 h after germination. (K) In vivo pollens growth. Pistils were collected, cleared, stained with Aniline Blue, and visualized with light microscopy 12 h after pollination. (TIF) [file pbio.1002571.s002.tif]

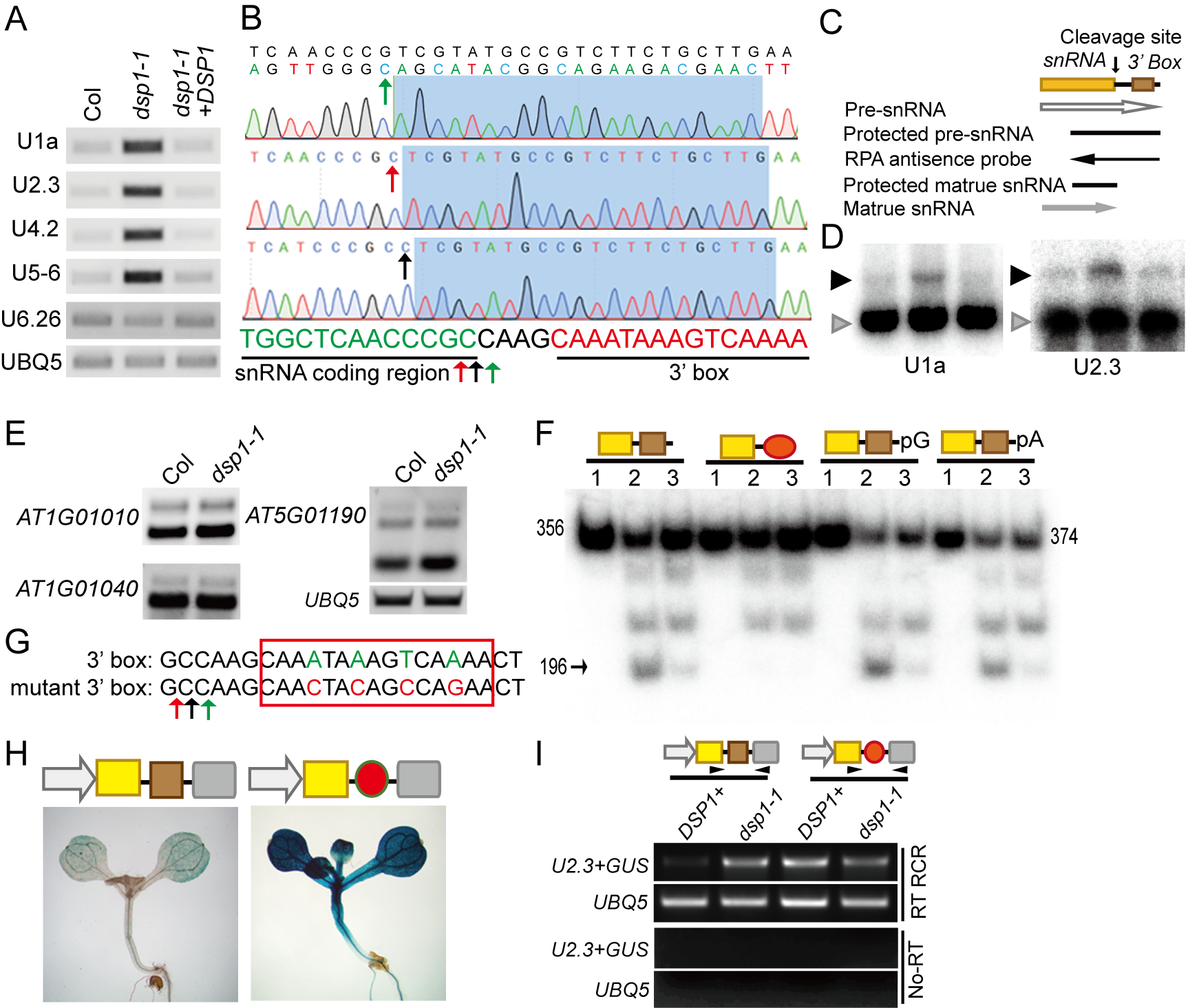

Supplement: S2 Fig — Related to Fig 2. (A) The abundance of various pre-snRNAs detected by RT-PCR. UBQ5 was amplified as a loading control. (B) Sequencing analyses of mature U2.3 snRNAs in dsp1-1. Three forms of mature U2.3 snRNAs were identified. Colored arrows indicate the 3′ terminal nucleotide of U2.3 snRNAs. The adapter sequence is covered with a blue box. (C) Diagram of snRNA probes used for RNase protection assay (RPA). The structure of pre-snRNA is shown on top. White and gray arrows represent primary and mature transcripts, respectively. Black arrow indicates antisense RPA probe. Grey and black lines indicate the protected fragments from RNase treatment. (D) The premature and mature U1a and U2.3 snRNAs detected by RPA. Ten micrograms of total RNA were incubated with [32P]-labeled RNA probe and treated by RNase T1 and RNase A. After reactions, RNAs were separated on a PAGE gel and detected with a PhosphorImager. Black arrow indicates pre-snRNAs. Grey arrow indicates mature snRNAs. (E) The transcripts of three protein-coding genes detected by RT-PCR. UBQ5 was amplified as a loading control. (F) In vitro processing of pre-U2, pre-U2m, pre-U2-pG, and pre-U2-pA RNAs. In vitro transcribed RNAs were [32P] labeled at 5′ end and processed in the nuclear protein extracts from Col for various times. pre-U2-pG: an 18-nt poly-G tail was added at the 3′ end of pre-U2.3 snRNA. pre-U2.3-pA: an 18-nt poly-A tail was added at the 3′ end of pre-U2.3 snRNA. After reaction, RNAs were resolved on PAGE gel and detected with a PhosphorImager. 1: reaction stopped at 0 min; 2: reaction stopped at 30 min; 3: reaction stopped at 60 min. (G) Sequence of the 3′ box and mutated 3′ box of the U2.3 gene. 3′ box is defined by the red rectangle. Green letter: nucleotide that was changed. Red letter: mutated nucleotide. The colored arrow indicates possible cleavage position. (H) GUS activities in Col harboring pU2::pre-U2-GUS, or pU2::pre-U2m-GUS. (I) The expression levels of pre-U2-GUS RNAs and pre-U2m-GUS RNAs [file pbio.1002571.s003.tif]

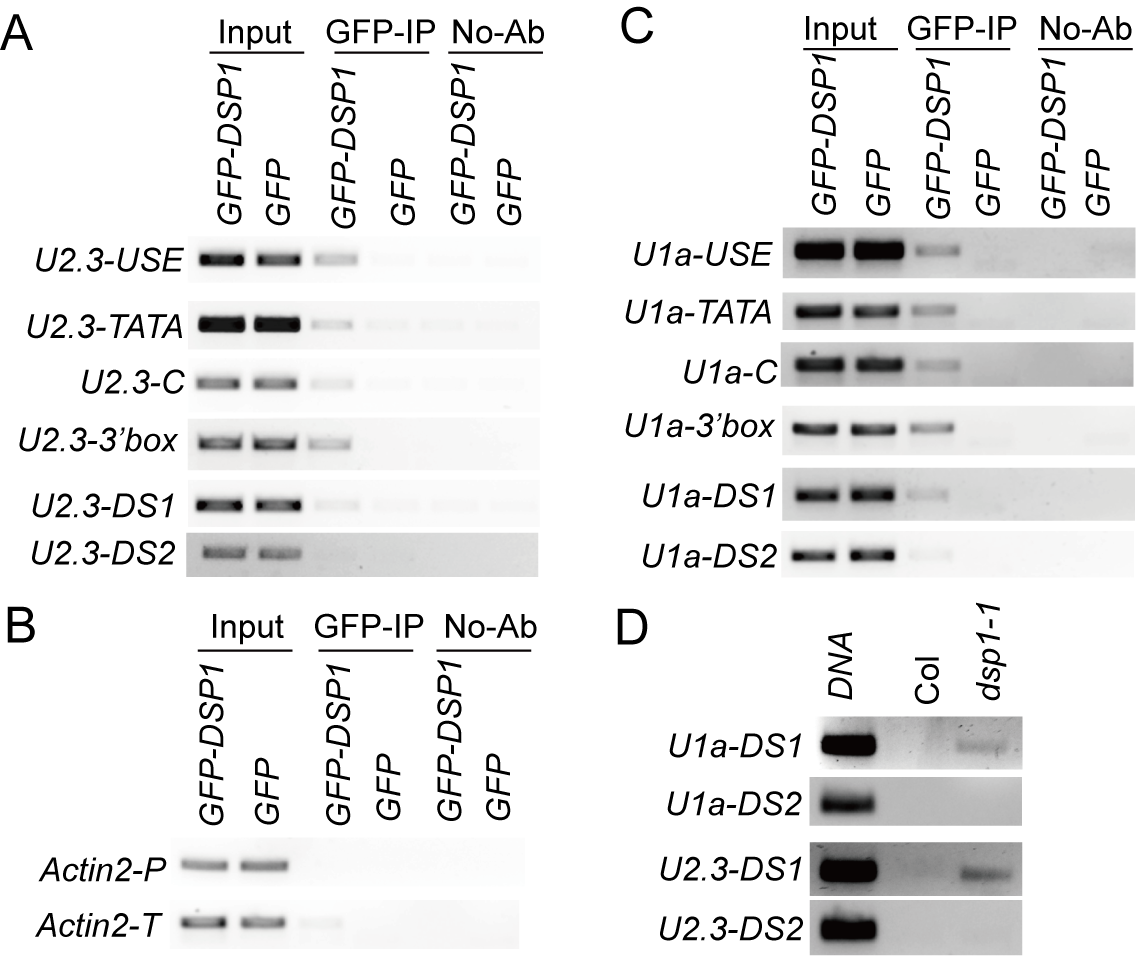

Supplement: S3 Fig — Related to Fig 3. (A–C) The occupancy DSP1 at the U2.3, U1a, and ACTIN2 loci detected by ChIP in transgenic plants harboring DSP1-GFP or GFP. PCR was used to analyze DNAs co-purified with DSP1-GPF or GFP. (D) Detection of the 3′ end transcript of U1a and U2.3 snRNAs in Col and dsp1-1 by RT-PCR. DS1 and DS2 localize downstream of the 3′ box of the U1a or U2.3 genes. PCR amplification of genomic DNA serves as a positive control. (TIF) [file pbio.1002571.s004.tif]

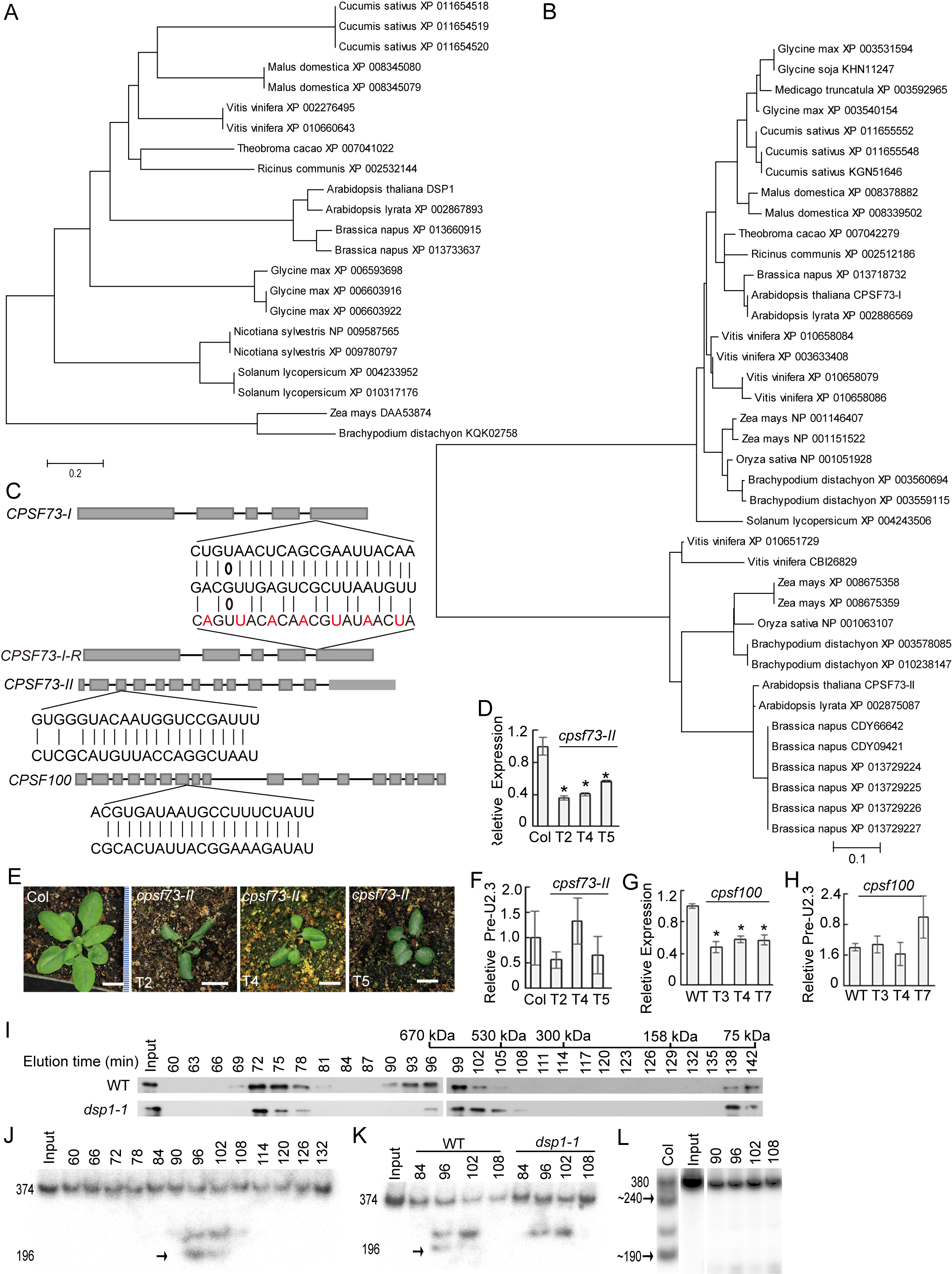

Supplement: S4 Fig — Related to Fig 4. (A) and (B) Phylogenetic analyses of DSP1 and CPSF73 homologs in plants. The full-length protein sequences were used to construct a Maximum Likelihood tree based on the Jones–Taylor–Thornton model. Scale bar represent the estimated number of substitution per site. (C) Schemes of artificial miRNAs targeting CPSF73-I, CPSF73-II, and CPSF100. The degree of pairing between the amiRNA and target gene is shown. The red letters indicate the mutated nucleotides in amiRNA target region of CPSF73-I. Solid line represents Watson–Crick pairing and a “0” indicates a G-U pairing. (D–F) The effect of amiRCPSF73-II on plant development and the transcript levels of CPSF73-II and pre-U2.3 snRNA. cpsf73-II: amiRCPSF73-II. The transcript levels of CPSF73-II or pre-U2.3 in amiRNA lines were detected by qRT-PCR, normalized to UBQ5 and compared with those in Col (value set as 1). **p < 0.01, *p < 0.05 (t test). (G) The transcript levels of CPSF100 in three amiRCPSF100 lines detected by qRT-PCR. cpsf100: amiRCPSF100. *p < 0.05 (t test). (H) The transcript levels of pre-U2.3 RNAs in three amiRCPSF100 lines detected by qRT-PCR. cpsf100: amiRCPSF100. (I) Gel filtration analysis of the CPSF73-I complex in Col and dsp1-1. Protein extracts from Col and dsp1-1 harboring GFP-CPSF73-I were separated by HPLC. Eluted fractions were separated by SDS–PAGE and detected by western blotting. Elution times are shown on the top of the picture. (J) In vitro processing of pre-U2.3 snRNAs by eluted proteins from Col. Elution times are shown on the top of picture. Reactions were stopped at 60 minutes. Arrow indicates mature snRNAs. (K) dsp1-1 reduced the snRNA processing activity of the CPSF73-I complex. (L) In vitro processing of RSB-3 using the CPSF73-I complex that acts on pre-snRNAs. Reaction using nuclear extracts from Col was used as positive control. RSB-3 represents the 3′ UTR of the Rubisco small subunit gene. (TIF) [file pbio.1002571.s005.tif]

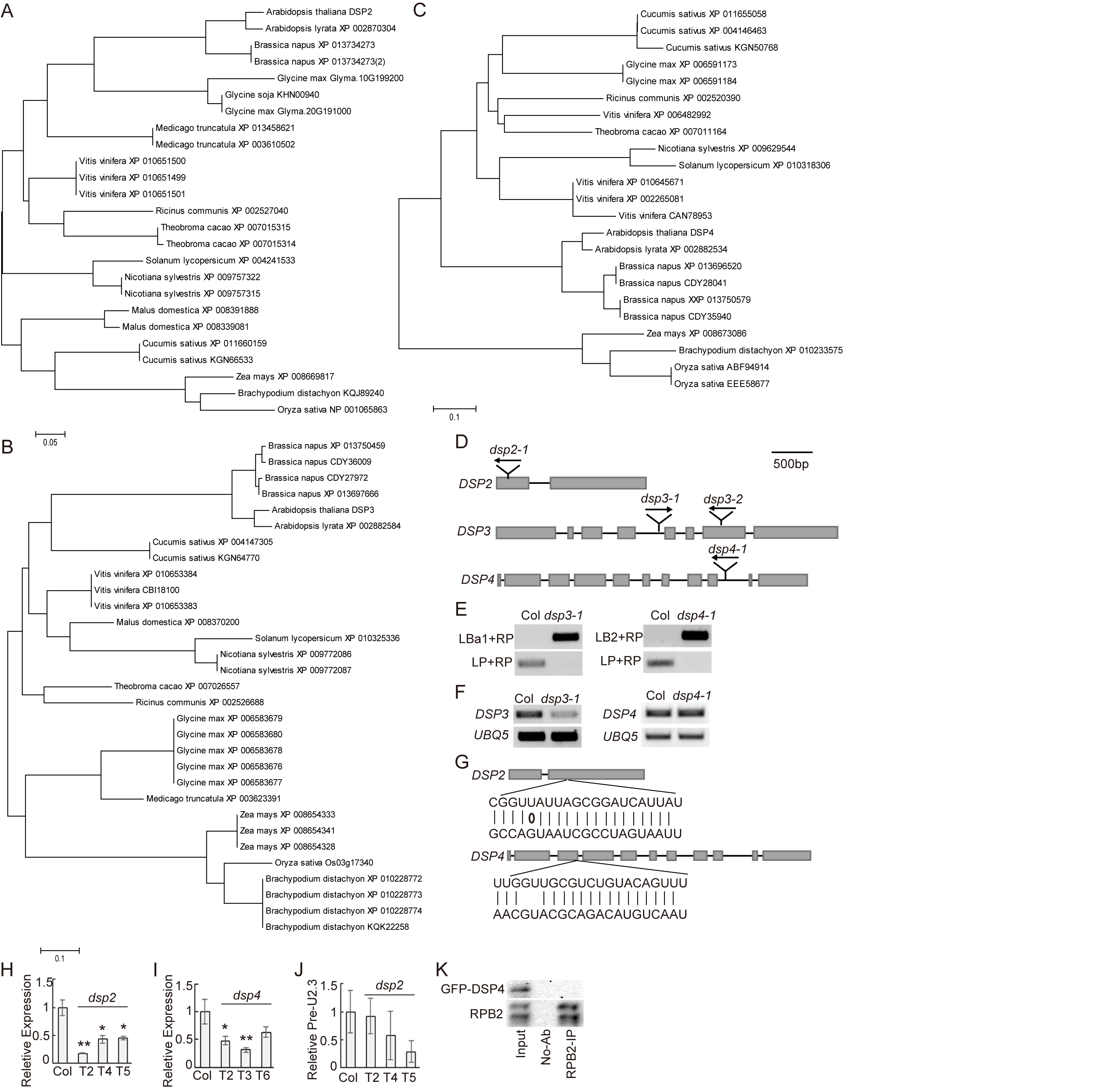

Supplement: S5 Fig — Related to Fig 5. (A–C) Phylogenetic analysis of DSP2, DSP3, and DSP4 in plants. The full-length protein sequences were used to construct a Maximum Likelihood tree based on the Jones–Taylor–Thornton model. Scale bar represents the estimated number of substitution per site. (D) Schemes showing the T-DNA insertion positions in DSP2, DSP3, and DSP4. (E) PCR analyses of T-DNA insertion in various genotypes. The primer combinations LP/RP and LBa1 (LB2)/RP are diagnostic for genes and the T-DNA flanking genomic DNA, respectively. (F) RT-PCR analysis of the DSP3 and DSP4 transcripts in their T-DNA insertion mutants. UBQ5 was amplified as a control. (G) Diagrams showing the artificial miRNAs targeting DSP2 or DSP4. Solid line indicates Watson–Crick pairing and a “0” defines a G-U pairing. (H) and (I) Transcript levels of DSP2 and DSP4 in their amiRNA lines. dsp2: amiRDSP2; dsp4: amiRDSP4. The abundance of DSP2 or DSP4 in the amiRNA lines was normalized to UBQ5 and compared with those in Col (set as 1). **p < 0.01, *p < 0.05 (t test). (J) The accumulation of pre-U2.3 snRNAs in amiRDSP2. dsp2: amiRDSP2. Transcript levels of pre-U2.3 snRNA were normalized to UBQ and compared with those in Col (value set as 1). **p < 0.01, *p < 0.05 (t test). (K) DSP4 does not interact with Pol II. IP was performed using Arabidopsis harboring the GFP-DSP4 transgene (TIF) [file pbio.1002571.s006.tif]

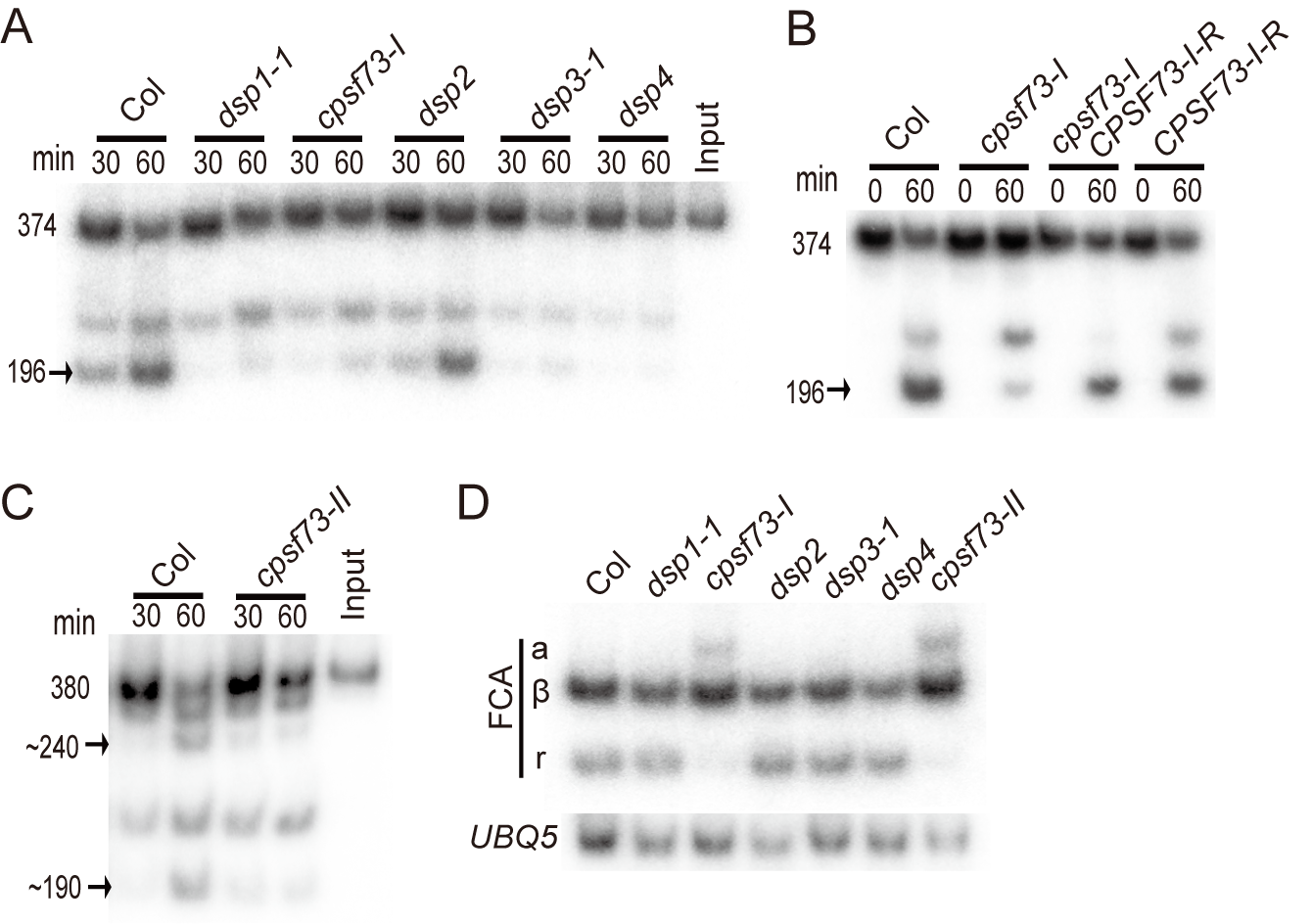

Supplement: S6 Fig — Related to Fig 5. (A) and (B) In vitro processing of pre-U2.3-pG snRNAs in the nuclear protein extracts from various genotypes. In vitro transcribed pre-U2.3-pG (pre-U2.3 snRNAs with poly G at 3′ end) were [32P] labeled at 5′ end and processed in the nuclear protein extracts from various genotypes. dsp2: amiRDSP2; dsp4: amiRDSP4; cpsf73-I: amiRCPSF73-I. (C) In vitro processing of RSB-3 in nuclear protein extracts from Col and cpsf73-II. cpsf73-II: amiRCPSF73-II. RSB-3: The 3′ UTR of the Rubisco small subunit gene. (D) The accumulation of FCA transcripts in various genotypes. FCA transcripts were detected by northern Blot. UBQ5 was probed as loading control. (TIF) [file pbio.1002571.s007.tif]

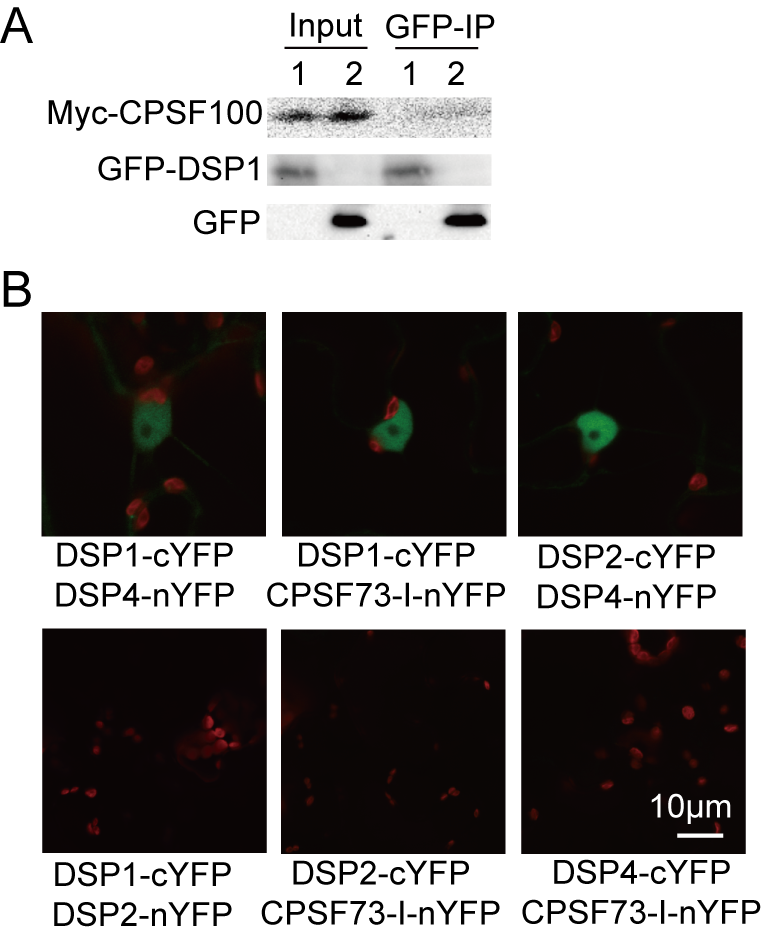

Supplement: S7 Fig — Related to Fig 6. (A) DSP1 does not interact with CPSF100. GFP-DSP1 (1) or GFP (2) was co-expressed with MYC-CPSF100 in N. benthamiana. Proteins detected by western blot were labeled on the left side of the picture. (B) BiFC analysis of the interactions among DSP1, DSP2, DSP4, and CPSF73-I. Respective pairs of cYFP and nYFP fused proteins were co-expressed in N. benthamiana leaves. Yellow fluorescence (green in image) signals were detected by confocal microscopy. Red fluorescence was from chlorophyll. Approximately 20 nuclei were examined for each pair, and an image is shown. (TIF) [file pbio.1002571.s008.tif]
