# Supplementary material for: snRNA 3′ End Processing by a CPSF73-Containing Complex Essential for Development in Arabidopsis
Source: PLoS Biol. 2016 Oct 25;14(10):e1002571. doi: 10.1371/journal.pbio.1002571 (PMC5079582; doi:10.1371/journal.pbio.1002571)
Supplement: S1 Table — Genetic assay of male transmission in dsp1-2 by reciprocal. (DOCX) [file pbio.1002571.s009.docx]

**S1 Table**

crosses.

| Parental genotype  **♀** X ♂ | Genotype of F1 plants | | | Transmission  Efficiency |
| --- | --- | --- | --- | --- |
|  | *DSP1/dsp1-2* | DSP1 | |  |
| *DSP1*/*dsp1-2* X *WT* | 76 | 84 | 76/84X100%=90.5% | |
| *WT* X *DSP1*/*dsp1-2* | 52 | 118 | 52/118X100%=44.1% | |
