# Supplementary material for: snRNA 3′ End Processing by a CPSF73-Containing Complex Essential for Development in Arabidopsis
Source: PLoS Biol. 2016 Oct 25;14(10):e1002571. doi: 10.1371/journal.pbio.1002571 (PMC5079582; doi:10.1371/journal.pbio.1002571)
Supplement: S2 Table — (DOCX) [file pbio.1002571.s010.docx]

| **S2 Table**  **Plasmids construction** | | | | | | | |  |  |  |
| --- | --- | --- | --- | --- | --- | --- | --- | --- | --- | --- |
| **Primer name** | | **Description** | | | | | | **Sequence 5' to 3'** |  |  |
| Pro-DSP1-TOPO-F | *DSP1* promoter cloning | | | | caccTCTAGAGGAGGAAGAAGAAGG | | | |  |  |
| Pro-DSP1-R | *DSP1* promoter cloning | | | | GGTTAGCACTTTTTGGCTTCTTAGGGCT | | | |  |  |
| DSP1-TOPO-F | *DSP1* gene cloning | | | | caccATGGAGAAGGTTTCAGCAGCT | | | |  |  |
| DSP1-R | *DSP1* gene cloning | | | | TGACGCTTTCTTAACCGTAAACAC | | | |  |  |
| CPSF73-I-TOPO-F | *CPSF73-I* gene cloning | | | | caccATGGCTTCTTCTTCTACTTCTC | | | |  |  |
| CPSF73-I-R | *CPSF73-I* gene cloning | | | | AGAAGCTGAGAGAGGGATTGG | | | |  |  |
| CPSF100-TOPO-F | *CPSF100* gene cloning | | | | caccATGGGTACTTCGGTGCAAG | | | |  |  |
| CPSF100-R | *CPSF100* gene cloning | | | | TAGAACTGAGAATAGAG | | | |  |  |
| DSP2-TOPO-F | *DSP2* gene cloning | | | | caccATGGAGAACCACTTTGTTAC | | | |  |  |
| DSP2-R | *DSP2* gene cloning | | | | CCGTGAGGGTCTTATAAACTC | | | |  |  |
| DSP4-TOPO-F | *DSP4* gene cloning | | | | caccATGGAATTGACATGTCTGAG | | | |  |  |
| DSP4-R | *DSP4* gene cloning | | | | ACCGTCAAGAACTCCATCAATC | | | |  |  |
| DSP3-TOPO-F | *DSP3* gene cloning | | | | caccATGACTTCATCAATGGAGGA | | | |  |  |
| DSP3-R | *DSP3* gene cloning | | | | TGAAACAAGAGAAAGGTGAATTTC | | | |  |  |
| U2-Pro-TOPO-F | U2.3 snRNA cloning | | | | caccTCCTGACTCCTGTTTACG | | | |  |  |
| U2-RNA-R | U2.3 snRNA cloning | | | | CTGCGTAACATATATAAATATCTCTG | | | |  |  |
| amiR-F | amiRNA cloning | | | | caccATACTACAAACGCCCCT | | | |  |  |
| amiR-R | amiRNA cloning | | | | GATTTAATCAACCGTCGA | | | |  |  |
| Red are complementary to the vector sequence for LR reaction | | | | | | | | |  |  |
|  | | |  | | | | |  |  |  |
| **Amplify template for in vitro transcription/translation** | | | | | | | |  |  |  |
| **Primer name** | | **Description** | | | | | | **Sequence 5' to 3'** |  |  |
| T7-Protein-F | *DSP3-Myc* | | | GCGAATTAATACGACTCACTATAGGGCTTAAGTATAAGGAGG | | | | |  |  |
| T7-DSP3-F | *DSP3-Myc* | | | GGCTTAAGTATAAGGAGGAAAAAATATGACTTCATCAATGGA | | | | |  |  |
| T7-protein-R | *DSP3-Myc* | | | AAACCCCTCCGTTTAGAGAGGGGTTATGCTAGTTA | | | | |  |  |
| T7-MYC-R | *DSP3-Myc* | | | GAGAGGGGTTATGCTAGTTAACCGTTCAAGTCTTCCTC | | | | |  |  |
| T7-U1-R | reversed U1a probe | | | TAATACGACTCACTATAGGGCTTTATTTAACA | | | | |  |  |
| U1-C-F | reversed U1a probe | | | GAGTGGCCTAGGCTAGTGACC | | | | |  |  |
| T7-U2-R | reversed U2.3 probe | | | TAATACGACTCACTATAGGGAAAACGTTATTTAAC | | | | |  |  |
| U2-C-F | reversed U2.3 probe | | | TCTCGGCCTTTTGGCTAAG | | | | | |  |
| T7-U2F | forward U2.3 probe | | | TAATACGACTCACTATAGGGATACCTTTCTCGGC | | | | |  |  |
| U2-RNA-R | forward U2.3 probe | | | CTGCGTAACATATATAAATATCTCTG | | | | |  |  |
| T7-AT5G38420-UTR-F | *AT5G38420* 3-UTR probe | | | TAATACGACTCACTATAGGGCTTAATCCCCT | | | | |  |  |
| AT5G38420-UTR-R | *AT5G38420* 3-UTR probe | | | GGTTTGATTGATTGCTTTCATGTG | | | | | | |
| Red are complementary to the gene sequence | | | | | | | | |  |  |
|  | | |  | | | | |  |  |  |
| **ChIP PCR** | | |  | | | | |  |  |  |
| **Primer name** | | | **Description** | | | | | **Sequence 5' to 3'** |  |  |
| U1-USE-F | | U1 USE | | | TGCACTTCAATGGGCCTAGAG | | | |  |  |
| U1-USE-R | | U1 USE | | | GCGATGTGGGACTCTGCAAAT | | | |  |  |
| U1-TATA-F | | U1 TATA | | | CTAAGACCTGAAAGAATG | | | |  |  |
| U1-TATA-R | | U1 TATA | | | AGTAATTTTCACAGACTGTC | | | |  |  |
| U1-C-F | | U1 Coding region | | | GAGTGGCCTAGGCTAGTGACC | | | |  |  |
| U1-C-R | | U1 Coding region | | | TGACGCAGGCTCTCCCACTT | | | |  |  |
| U1-3'box-F | | U1 3' box | | | CCCTACCATTCTTTTTACATG | | | |  |  |
| U1-3'box-R | | U1 3' box | | | TGACGCAGGCTCTCCCACTT | | | |  |  |
| U1-DS1-F | | U1 down-stream 1 | | | | GTCCTCAGCAGTCAGCAACA | | |  |  |
| U1-DS1-R | | U1 down-stream 1 | | | | TGTTGCCGAGAACTTGTAAGC | | |  |  |
| U1-DS2-F | | U1 down-stream 2 | | | | AGGAAGAAGGTCGCTGTTGG | | |  |  |
| U1-DS2-R | | U1 down-stream 2 | | | | CGCGTGAATATCGGTCCAGA | | |  |  |
| Actin2-Pro-F | | *Actin2* promoter | | | | CCAACCACGACGACGACTAA | | |  |  |
| Actin2-Pro-R | | *Actin2* promoter | | | | CAGACAGCCTTTTCCCTCGT | | |  |  |
| U2-USE-F | | U2 USE | | | | GAGATACGTTACAGCATCTCG | | |  |  |
| U2-USE-R | | U2 USE | | | | CAGCCATAAGAATGTTCTCA | | |  |  |
| U2-TATA-F | | U2 TATA | | | | ACCTCCTGACGATGCAATGAG | | |  |  |
| U2-TATA-R | | U2 TATA | | | | AAGGTATGATTTGGACGATGG | | |  |  |
| U2-C-F | | U2 Coding region | | | | TCTCGGCCTTTTGGCTAAG | | |  |  |
| U2-C-R | | U2 Coding region | | | | AGTGCAACGCATGGGCGA | | |  |  |
| U2-3'box-F | | U2 3' box | | | | CAACCCGCCAAGCAAATAAAGTC | | |  |  |
| U2-3'box-R | | U2 3' box | | | | CGAAGTTTTCTCCATTCAC | | |  |  |
| U1-DS1-F | | U2 down-stream 1 | | | | AAATTCCCGAGCTTTGCGAC | | |  |  |
| U1-DS1-R | | U2 down-stream 1 | | | | CGGCCAGTGCTCATCAAGAA | | |  |  |
| U1-DS2-F | | U2 down-stream 2 | | | | ATGGTCCACTAACCAAAGCGA | | |  |  |
| U1-DS2-R | | U2 down-stream 2 | | | | | TGAGTTCTTGCTGCTACCTGT | |  |  |
|  | | |  | | | | |  |  |  |
| **qPCR** | | |  | | | | |  |  |  |
| **Primer name** | | **Description** | | | | | | **Sequence 5' to 3'** |  |  |
| CPSF73-II-RT-F | | *CPSF73-II* | | | | | TGTGGGATGCATATGGGCTG | |  |  |
| CPSF73-II-RT-R | | *CPSF73-II* | | | | | TGAATAGCTCCTCCTCGCCT | |  |  |
| CPSF73-I-RT-F | | *CPSF73-I* | | | | | TGGAGGTCTTCAAAGCGGTC | |  |  |
| CPSF73-I-RT-R | | *CPSF73-I* | | | | | CGTCTTGGCCAGTGTACCTT | |  |  |
| CPSF100-RT-F | | *CPSF100* | | | | | AGCTTGGGTGGATTCCGAAG | |  |  |
| CPSF100-RT-R | | *CPSF100* | | | | | GAACACCCTTGCTCGACAGA | |  |  |
| DSP2-RT-F | | *DSP2* | | | | | ACTCCCAGACCCACAAGAGT | |  |  |
| DSP2-RT-R | | *DSP2* | | | | | ACAAACTAATTCCAATTCGTCGC | |  |  |
| DSP3-RT-F | | *DSP3* | | | | | CTCGGCTGTGGCTACTTAGG | |  |  |
| DSP3-RT-R | | *DSP3* | | | | | GGAGTGGAAGGAGTCAACCG | |  |  |
| DSP4-RT-F | | *DSP4* | | | | | TGCTCGGATTGCCATTCCTT | |  |  |
| DSP4-RT-R | | *DSP4* | | | | | CACTCTCGCCGAAGACAACT | |  |  |
| DSP1-RT-F | | *DSP1* | | | | | GCTCTGTTTGCTGCAGCTTGC | |  |  |
| DSP1-RT-R | | *DSP1* | | | | | GATCTTGAAGCAAGCTTAGTC | |  |  |
| U1a-C-F | | pre-U1a snRNA | | | | | GAGTGGCCTAGGCTAGTGACC | |  |  |
| U1a-E-R | | pre-U1a snRNA | | | | | ATTTAACAAAGGTTTCATGT | |  |  |
| U4.2-C-F | | pre-U4.2 snRNA | | | | | GCAATGACGCAGCTAATGAGGT | |  |  |
| U4.2-E-R | | pre-U4.2 snRNA | | | | | TTCTAAATTTCAAAACTCGA | |  |  |
| U5.6-C-F | | pre-U5.6 snRNA | | | | | ACGCAGCCATGTGGTGAGCA | |  |  |
| U5.6-E-R | | pre-U5.6 snRNA | | | | | CTCAGATACTTCGAGACCAA | |  |  |
| U6.26-F | | pre-U6.26 snRNA | | | | | GTCCCTTAGGGGACATCCGA | |  |  |
| U6.26-E-R | | pre-U6.26 snRNA | | | | | CGCCGAAGAACAGAGGAAGAA | |  |  |
| U2-C-F | | pre-U2.3 snRNA | | | | | TCTCGGCCTTTTGGCTAAG | |  |  |
| U2-R-2 | | pre-U2.3 snRNA | | | | | CGAAGTTTTCTCCATTCAC | |  |  |
|  | | |  | | | | |  |  |  |
| **Genotyping** | | |  | | | | |  |  |  |
| **Primer name** | | **Description** | | | | | | **Sequence 5' to 3'** |  |  |
| SALK_036641-LP | | *dsp1-1* | | | | | TTCAGTTAAAGCCGTTGAAGC | |  |  |
| SALK_036641-RP | | *dsp1-1* | | | | | TCGCAAGATGGTAACAACAGC | |  |  |
| CS16199-LP | | *dsp1-2* | | | | | GTAGACCCCAATGTCTGCGA | |  |  |
| CS16199-RP | | *dsp1-2* | | | | | TGACGCTTTCTTAACCGTAAACA | |  |  |
| CS848944-LP | | *dsp2-1* | | | | | TTCCATCTCAGGAGTGATTCG | |  |  |
| CS848944-RP | | *dsp2-1* | | | | | AGACAATTGCAGGATCACACC | |  |  |
| SALK_089544-LP | | *dsp3-2* | | | | | ACAAATTGGCAAGTTTCATCG | |  |  |
| SALK_089544-RP | | *dsp3-2* | | | | | CTGACGCTGATAATCTCAGCC | |  |  |
| SALK_005904-LP | | *dsp4-1* | | | | | TCGATGGATCCTTTTATTCCC | |  |  |
| SALK_005904-RP | | *dsp4-1* | | | | | ACCATGCATCGTATTTGCTTC | |  |  |
| SALK_086160-LP | | *dsp3-1* | | | | | CAGGAAACTTATATGCCTGC | |  |  |
| SALK_086160-RP | | *dsp3-1* | | | | | AGTTGCTAACTCCTGTTTGC | |  |  |
|  | | |  | | | | |  |  |  |
| **snRNA cleavage site assay** | | | | | | | |  |  |  |
| **Primer name** | | | **Description** | | | | | **Sequence 5' to 3'** |  |  |
| Adaptor-rt-primer | | target to RNA adaptor | | | | | CAAGCAGAAGACGGCATACGA | |  |  |
| 3' RNA adaptor | | RNA ligation | | | | | P-UCGUAUGCCGUCUUCUGCUUG-UidT | |  |  |
| U2-nest1 | | U2.3 snRNA | | | | | ATACCTTTCTCGGCCTTTTGGC | |  |  |
| U2-nest2 | | U2.3 snRNA | | | | | TCTCGGCCTTTTGGCTAAG | |  |  |
|  | | |  | | | | |  |  |  |
| **Site-Directed Mutagenesis** | | | | | | | |  |  |  |
| **Primer name** | | | **Description** | | | | | **Sequence 5' to 3'** |  |  |
| U2-3'box-mut-F | | 3' box mutation | | | | | AGCAACTACAGCCAGAACTTTTTAAACC | |  |  |
| U2-3'box-mut-R | | 3' box mutation | | | | | AGTTCTGGCTGTAGTTGCTTGGCGGGT | |  |  |
| Red are mutated site | | | | | | | |  |  |  |
