# Supplementary material for: snRNA 3′ End Processing by a CPSF73-Containing Complex Essential for Development in Arabidopsis
Source: PLoS Biol. 2016 Oct 25;14(10):e1002571. doi: 10.1371/journal.pbio.1002571 (PMC5079582; doi:10.1371/journal.pbio.1002571)
Supplement: S1 Text — (DOCX) [file pbio.1002571.s011.docx]

**Supplemental Experimental Procedures**

**U2 snRNA cleavage site analysis**

RNAs, RNAs extracted from *dsp1-1* were dephosphorylated and ligated to a 3′ RNA adaptor. The ligation products were used as templates for reverse transcription using a primer recognizing the adaptor. snRNAs are then PCR amplified and cloned for sequencing. Primers used are listed in S2 Table.

**Artificial miRNA construction**

To generate amiRNA vectors, DNA fragments containing the *MIR164A* backbone, in which the miR164/miR164* was replaced with an amiRNA/amiRNA*, were synthesized and cloned into PMDC32. Various amiRNAs were designed using the Web MicroRNA Designer (WMD3) [1]

**BiFC assay**

*DSP1*, *DSP2*, *DSP4* and *CPSF73-I* were cloned into the pEarleyGate201-YN or pEarleyGate202-YC vector [2], respectively. The paired constructs were introduced into *N. benthamiana* leaf through infiltration. The YFP signal was observed with a confocal microscope.

References:

1. Schwab R, Ossowski S, Riester M, Warthmann N, Weigel D. Highly specific gene silencing by artificial microRNAs in Arabidopsis. Plant Cell. 2006;18(5):1121-33. doi: 10.1105/tpc.105.039834. PubMed PMID: 16531494; PubMed Central PMCID: PMCPMC1456875.

2. Lu Q, Tang X, Tian G, Wang F, Liu K, Nguyen V, et al. Arabidopsis homolog of the yeast TREX-2 mRNA export complex: components and anchoring nucleoporin. Plant J. 2010;61(2):259-70. doi: 10.1111/j.1365-313X.2009.04048.x. PubMed PMID: 19843313.
